# Supplementary material for: Behavior Stability and Individual Differences in Pavlovian Extended Conditioning
Source: Front Psychol. 2020 Apr 22;11:612. doi: 10.3389/fpsyg.2020.00612 (PMC7189120; doi:10.3389/fpsyg.2020.00612)
Supplement: Supplementary file 1 [file Data_Sheet_1.pdf]

Supplementary material: Behavior stability and individual differences in Pavlovian extended conditioning

1 DESCRIPTION

This Supplementary Material includes additional theoretical considerations, data and figures that may be useful to the reader, but not essential to understand the main article. Among the theoretical aspects, we introduce an independent model characterized by the presence of a stochastic noise of cognitive origin. We also consider a model where response fluctuations are governed by the laws of quantum mechanics. Data are compatible with this quantum extension, although experimental uncertainties dominate the result. On the other hand, data do not favor models with a long-memory effect or where response variability is solely described by a random fractal.

Several technical remarks and figures regarding our experiment are given in section 2. Section 3 presents the details of the DOM (RW model with friction) as well as relevant applications of the dynamical approach for future checks of the theory, especially to the multi-cue RW and Mackintosh models. The basics of spectral analysis and data figures are discussed in section 4. In section 5, we consider scenarios which are excluded, or at least not confirmed, by the data: one encoding a long-memory effect and one where response variability is described by a random fractal. A predictive theory where random fluctuations of the subject response are described by the mathematics of quantum mechanics is discussed in section 6. Data neither rule out nor confirm the quantum model and one cannot discard the interpretation of response variability as quantum fluctuations.

2 SUPPLEMENTARY MATERIAL ABOUT OUR EXPERIMENT

2.1 Description of the experiment

2.1.1 Subjects

32 male Wistar Han non-naïve rats (Charles River Laboratories) were used. These rats proceeded from three different operant-conditioning experiments conducted in the same laboratory, but they had no prior overt training in Pavlovian conditioning nor they had been trained in the same apparatus. The characteristics of previous experiments were such that any significant influence on the present work is most unlikely.

Four subjects began the experiment with 43 weeks of age and ended 15 weeks later. 18 subjects started with 39 weeks and 10 subjects with 21 weeks; all of them ended 10 weeks later. The average age at the beginning and end of the experiment was of, respectively, 33.9 ± 8.8 and 44.5 ± 9.5 weeks.

The subjects were kept in individual identical cages of size $19\,(\mathrm{h}) \times 23.5\,(\mathrm{w}) \times 35.5\,(\mathrm{l})$ cm (non-enriched environment) with unrestricted water supply and a restricted diet of food to maintain 100% of their theoretical body weight. The average theoretical weight was of 418 ± 25 g. In theory, therefore, animals were neither food nor water deprived.

2.1.2 Materials

Four identical conditioning boxes were used. Each experimental box was 35.5 cm in length by 29 cm in height and 24.5 cm in depth, and was enclosed within a sound-attenuating chest, equipped with a fan, which provided ventilation and masking noise, a fluorescent lamp (20 W), which served as houselight, and a window for observation in the frontal part. The front panel of each experimental chamber was of aluminum, the posterior panel was of black metal, and the remaining walls were made of transparent plexiglass.

Two syringes connected to water pumps were installed in the back of the front panel. The water-pump device was designed at the Department of Basic Psychology I at UNED and made by CIBERTEC (Madrid, Spain). It consisted in a 24 V DC pinch-type electrovalve by ASCO placed between a 60 ml depot and a capillary exit, connected by a silicon tube (1.74 mm external diameter, approximately 10 cm length). The exit was connected to a metal water tube, 5 mm of diameter, which protruded 4 cm into the experimental chamber. Each water tube was located at the sides of the front panel (10 cm from the food magazine).

Throughout the experiment, only one tube was active, the other one being inert. The contact of the tongue of the rats with the water tube resulted in the activation of the pump during 0.1 s, whenever this response was reinforced; otherwise licks were recorded and the liquid was not delivered.

As for the US, we prepared two solutions of water and saccharin (sodium saccharin hydrate, Sigma-Aldrich, St. Louis, MO) at 0.1% (1 g/l) and 0.2% (2 g/l), respectively, less than aversive concentrations ($\gtrsim 0.3\%$), much less than toxic dosage (14.2 g/Kg for rats), and for a much shorter time inducing liver inflammation (0.3 g/l for six months for mice; Bian et al., 2017); see also Fujita et al. (2009). The 0.1% concentration was strong enough for subjects to discriminate it from plain water (saccharin concentrations as low as 0.05% have been used in the literature; see Swithers and Hall, 1994). A 0.2% concentration was successfully used in the literature as an appetitive stimulus (Bernal et al., 2008; Sclafani and Ackroff, 1994). The solutions were conserved in two one-liter jars in a laboratory fridge at constant temperature and were freshly remade weekly according to consumption by the subjects during the experimental sessions. The jars were taken out of the fridge to reach laboratory temperature (21°C) before the start of the first session of the day.

Experimental and control programs were written in PASCAL language and executed in the boxes via MED-PC IV® (Med Associates Inc., Fairfax, VT), which also recorded the responses. Data analysis was performed with Microsoft Excel and Wolfram Mathematica.

2.2 Technical remarks

We present some general remarks about the experimental design of our experiment.

- In Eq. (6), we defined unconditioned responses as those occurring in the presence of the US and assumed that the number of US is equivalent to the number of unconditioned responses. However, this assumption does not account for the following situation. It could be that, sometimes, the subject did not lick upon delivery of the US and that it licked instead afterwards, in the absence of the US (vacuum licking). Although a single drop stayed clung to the dispenser, the short delivery of two US could make the merger of the first and second drop fall before the animal could lick, at which point vacuum licking would take place. All these fine details are not important for the final interpretation of the results, because replacing (6) with a different operational definition of v would lead to very small quantitative differences. For example, we checked that considering, in alternative, the number of licks during the CS (not discounting the number of US), the number of licks per reinforcer (number of licks during CS)/(number of US), or the number of CS licks minus the number of post-CS licks does not change the plots qualitatively, and response variability remains at the same levels. Therefore, it is in general reasonable to assume that the associative strength is manifested in the overall behavior.
- Unrestricted access to water away from experimental sessions and a controlled diet set at 100% of the theoretical body weight guaranteed that the response of the subjects was not driven by either thirst or hunger. This was done to isolate purely associative effects beyond the RW model.
- The choice of a tone was made to avoid sign tracking and reduce alternative activities inside the box.
- Randomization of the inter-trial interval prevents the subject to use the latter as a predictor of the US, thus guaranteeing that the association is made between the CS and the US only.
- A conditioning is simultaneous when the US is always presented when the CS is on and the CS is presented when the US is on. This definition is similar to our experimental design, where the CS started and then the US came, finishing before the end of the CS. This design (US delivered during the CS instead of after) was forced upon us by some unavoidable technical characteristics of the available conditioning boxes. When the electrovalve open to release the drop of saccharin solution, the mechanism produces a click sound. We checked in a pilot experiment with delayed conditioning that rats are, in general, much more sensitive to sudden noises than continuous ones, and that the click sound of the electrovalve overshadowed the tone. While in the pilot experiment the subjects waited for the click to lick, and licked only enough to collect the US, in the actual experiment they waited for the auditory CS, since they could only predict that the electrovalve would click during the tone. Qualitative observations showed that the rats licked also before the click, and that overall they licked much more than in the pilot experiment. As we will see below, and as already established nowadays in several preparations, simultaneous conditioning is as effective as the delayed one. At any rate, it should be noted that our preparation is simultaneous only technically speaking, but in fact it is closer

to the more common delayed conditioning, since the CS always begins before any US is delivered. The Rescorla–Wagner model certainly accommodates this situation.

- The present design allows one to exclude behavioral changes due to non-associative factors such as satiation and fatigue. The subjects had daily access only to a very limited amount of reinforcer (about 100 drops per session), much lower than the quantity they can assume in a session of the same duration when free access is granted (several thousands of drops, as we checked in a pilot experiment conducted with a different set of animals).
- We checked explicitly that week-end or bank-holiday breaks did not have any impact on the subjects' performance, nor did variations of the time of the day at which sessions were executed. In a pilot experiment conducted with other rats, we checked that inter-session intervals below 40 minutes could have an effect on the response, hence the conservative lower bound of 90 minutes.

Also, concerning the animals:

- We found no correlation between the starting or final age of the subjects and their response. This excludes spurious effects due to the subjects not coming from the same batch.
- The data of one rat (subject 1-8) were eliminated from the analysis due to its poor health.

2.3 Data analysis

Figures S1 and S2 show the average of the raw (non-normalized) data for all groups. Notice that we did not subtract the US in the pre-CS, CS, and post-CS trendlines of control data, since they are equally affected by the unconditioned response (the US are evenly distributed in the whole session).

Using the RW model (Eq. (5) of the main paper) on the raw averaged data and employing the least squares method, we obtain the fits (**Figure S1**):

$$\text{Group 1:}\qquad \lambda = 540.1 \pm 7.1\,,\quad \alpha\beta = 0.129 \pm 0.011\,,$$
$$r^2 = 0.99\,,\quad \sigma = 55.8\,, \tag{S1a}$$
$$\text{Group 2:}\qquad \lambda = 687.6 \pm 8.1\,,\quad \alpha\beta = 0.182 \pm 0.017\,,$$
$$r^2 = 0.99\,,\quad \sigma = 68.0\,, \tag{S1b}$$

where r^2 is the coefficient of determination and σ is the estimated standard deviation of the fit.

We would like to comment on the fact that the raw average presented in **Figure S1** hides a caveat. Assuming the RW model for the time being, each subject has a particular asymptote λ and a learning rate $\alpha\beta$, with great variability among different rats. Therefore, one might doubt about the legitimacy of averaging the data without taking these differences into account. This point was discussed in the early literature and especially acknowledged by Sidman (1952), after which it has gone almost forgotten (but see Gallistel, 2012; Gallistel et al., 2004) because, after all, average data do turn out to be useful in a number of experimental situations. Nevertheless, we tackle the issue explicitly again, with some elementary but not-often-used techniques.

Let us then normalize the data *before* averaging. We do so with two independent methods that will yield the same result. The first method ("averaging procedure") is model-independent but assumes that there exists an asymptote of learning. To determine it, we remove the first and last 15 to 20 data points and make a linear regression of the remainder. As a general observation, the transient acquisition phase occupied the first 15 to 20 sessions, while in some cases the last 10 sessions were not as stable as intermediate ones. Thus, we took different intervals: sessions 16-90, 16-80, 16-70, 21-90, 21-80, and 21-70. The chosen interval is bases on a qualitative observation of the data and we selected the one giving the line with slope closest to zero within one standard deviation. Once selected the session range, we took the average of the data (linear regression with zero slope). The result is reported in **Table S1**.

The second method ("best-fit procedure") consists in assuming the RW model and use it to fit the individual data. For each subjects, a nonlinear regression yields an estimate for λ (**Table S1**) and $\alpha\beta$. As one can see, the values of λ obtained by both methods are very similar (the estimated standard deviation of the fits ranges from 80 to 240 licks, much larger than the difference $|\lambda_{\text{average}} - \lambda_{\text{best fit}}|$) and, in fact, they have a linear correlation with $r^2 \approx 0.98$ both in Group 1 and Group 2 (**Figure S3**). Therefore, the

determination of λ for each subject is quite robust and one can choose either method. In what follows, we take the values obtained by the nonlinear fit (last row of **Table S1**). Note that the value of the average λ for each Group is close to the one obtained from the best fit of the raw average data via the RW model, Eq. (S1), but the associated standard deviation is much larger. Group 1: $\langle\lambda_{\text{average}}\rangle = 542 \pm 209$, $\langle\lambda_{\text{best fit}}\rangle = 554 \pm 222$; Group 2: $\langle\lambda_{\text{average}}\rangle = 687 \pm 337$, $\langle\lambda_{\text{best fit}}\rangle = 727 \pm 369$. This error is a measure of the dispersion of the λ estimates around the average within each group, while the estimated standard deviation σ in Eq. (S1) is the mean dispersion of the data with respect to the theoretical curve.

Figures S4–S10 show individual subjects' data and their respective RW fits.

Regarding the discussion of the results, we mention that other possible effects of overtraining not considered in the main paper can be looked for in the long-term trend of post-CS data. A linear regression of post-CS normalized data of Group 1 yields a slightly negative slope -0.0010 ± 0.0002 and an intercept 0.41 ± 0.01 (the post-CS response is approximately 40% the response during the CS), while for Group 2 the slope is -0.0006 ± 0.0002 and the intercept 0.40 ± 0.01. In both cases, the "inertia" after the tone tends to decrease, but at a low rate.

A subtle point about averaging is also worth being covered for the sake of rigorousness. We have seen that averaging of normalized data is not very different from averaging of raw data, apart from a decrease of error bars. However, one might still question the validity of the normalized averaging in the acquisition phase, due to the fact that the acquisition rate $\alpha\beta$ can be very different from subject to subject (**Table S1**). Some rats reached the asymptote of learning very quickly, while others did so only towards the end of the experiment. Therefore, averaging over raw or normalized data does not take into account variability in the acquisition rate. To check whether this introduces unwanted artifacts, we can rescale the "time" of each rat to normalize also the acquisition rate $\alpha\beta$. To illustrate the point, assume that the RW model

$$v_{\text{excit}}(t) = \lambda\left(1 - e^{-\alpha\beta t}\right) \tag{S2}$$

holds and consider two subjects with learning curve $v_1(t) = \lambda_1[1 - \exp(-\alpha_1\beta_1 t)]$ and $v_2(t) = \lambda_2[1 - \exp(-\alpha_2\beta_2 t)]$. Normalizing the asymptote yields two curves $v_1(t)\lambda_1 = 1 - \exp(-\alpha_1\beta_1 t)$ and $v_2(t)/\lambda_2 = 1 - \exp(-\alpha_2\beta_2 t)$ that differ only in the learning rate. Calling $\tilde{t}_1 := \alpha_1\beta_1 t$ and $\tilde{t}_2 := \alpha_2\beta_2 t$, one ends up with a single profile $w(\tilde{t}) = 1 - \exp(-\tilde{t})$ evaluated at two different "times" $\tilde{t}_1$ and $\tilde{t}_2$. Doing this for all subjects, we obtained the cloud of points shown in Fig. S11. Since we used the RW model on individual data sets to normalize the asymptote and the learning rate, it should come as no surprise that this cloud of points is fit by the RW model with good accuracy, albeit data are rather dispersed (Group 1: $\lambda = 1.00 \pm 0.02$, $\alpha\beta = 1.00 \pm 0.10$, $r^2 = 0.88$, $\sigma = 0.34$; Group 2: $\lambda = 1.00 \pm 0.01$, $\alpha\beta = 1.00 \pm 0.07$, $r^2 = 0.92$, $\sigma = 0.27$).

2.4 Data interpretation

We present the full argument leading to the conclusion of the third bullet in section 4.3.2 of the main paper.

First of all, the partial reinforced schedule in PR groups is deterministic (one reinforcement every three trials). This is the reason why we could apply the RW model of continuous reinforcement also to these groups. The set of (nonreinforced trial 1) + (nonreinforced trial 2) + (reinforced trial 3) is seen as a unit by the model, so that three such trials of 10 s each (PR10 group) are similar, as a unit, to one trial of 30 s with one US (CR30 group), although they differ in the number of CS presented (three times as many in the PR10 group). Therefore, one would expect to find a similar behavioral trend in average in these two groups, which is what we observed: the (not great) majority of subjects follows a monotonic learning curve. The percentage of subjects following a stable (i.e., monotonic RW) behavior is further leveled between these two groups and with respect to subjects with unstable (i.e., oscillatory or random) behavior when we consider subjects whose response was so erratic that we could not find a fit among the available models. Then, the score stable-unstable is set to 50% – 50% in CR30 and to 56% – 44% in PR10. With respect to these two groups, PR30 gives rise to a less stable (more oscillatory fits) but less erratic (no failed fits) behavior, since there are longer nonreinforced trials interspersed between reinforced ones. This agrees with the above findings: the (great) majority of PR30 subjects display oscillations in their learning curve. Finally, regarding group CR10, subjects are reinforced as many times as, but on a shorter schedule than, in any other group. In average, this results in a slightly more stable behavior than PR30 (see table on

previous page), but in a less stable behavior than CR30. We close the loop and get confirmation of this when looking at the PR10 data: it has more erratic subjects than CR10 and PR30. Thus, it seems that *longer trials stabilize the behavior, but a partial reinforcement schedule destabilizes it.*

3 DYNAMICAL MODELS

3.1 Dynamical RW model with friction

Taking the time derivative of $\dot{v} = \alpha\beta(\lambda - v)$, Eq. (2) of the main paper,we obtain

$$\ddot{v} + 2\alpha\beta\dot{v} + (\alpha\beta)^2(v - \lambda) = 0\,. \tag{S3}$$

This equation can be derived from the action[1]

$$S_{\rm RW} = \int_0^T dt\,\tilde{\mathcal{L}}_{\rm RW}\,, \tag{S4}$$

where the Lagrangian $\mathcal{L}_{\rm RW}$ reads

$$\tilde{\mathcal{L}}_{\rm RW} = e^{2\alpha\beta t}\mathcal{L}_{\rm RW}\,, \qquad \mathcal{L}_{\rm RW} = \frac{\dot{v}^2}{2} - U(v)\,, \tag{S5a}$$

$$U(v) = \frac{(\alpha\beta)^2}{2}(v - \lambda)^2\,. \tag{S5b}$$

Let us explain all symbols and the procedure in detail. t is time and the integral runs from some initial time conventionally set to $t = 0$ to the time $t = T$ when the experiment ends. The function $v = v(t)$ is the association strength related to one CS. The ideal situation of maximal learning corresponds to $T = +\infty$. For instance, at the beginning of an excitatory conditioning the association strength will be $v(0) = 0$ and its velocity $\dot{v}(0) \neq 0$, while at arbitrarily large times $v(+\infty) = \lambda > 0$ (some asymptote determined by the magnitude of the US) and $\dot{v}(+\infty) = 0$.

In physics terminology, Eq. (S5) corresponds to a nonrelativistic, classical particle with *kinetic energy* $\dot{v}^2/2 \geqslant 0$ and *potential energy* $U(v)$. According to the variational principle in classical mechanics, the dynamical equation for a particle following a trajectory $x(t)$ is obtained by varying an action $S[x]$ with respect to a small fluctuation δx of the degree of freedom $x(t)$ and imposing that the action is stationary against this fluctuation ($\delta S = 0$) when the latter vanishes at the boundary of the integration domain, $\delta x(0) = 0 = \delta x(T)$. This means that the motion $x(t)$ solving the dynamics minimizes the path from point $x(0)$ to point $x(T)$. For this reason, the variational principle $\delta S[v]/\delta v = 0$ (Eq. (7) of the main paper) is also called the principle of least action. For an action $S[v]$ depending on one degree of freedom v, the equation of motion $\delta S[v]/\delta v = 0$ is equivalent to the Euler–Lagrange equation

$$\frac{d}{dt}\frac{\partial\tilde{\mathcal{L}}}{\partial\dot{v}} - \frac{\partial\tilde{\mathcal{L}}}{\partial v} = 0\,. \tag{S6}$$

It is easy to check that Eq. (S3) is given by (S6) when applied to (S5). For a generic potential, the equation of motion reads $\ddot{v} + 2\alpha\beta\dot{v} + U'(v) = 0$, where the prime is a derivative with respect to v and the *friction* term $2\alpha\beta\dot{v}$ is generated by the prefactor $\exp(2\alpha\beta t)$ in the action. Friction terms can arise in very different contexts in physics. One is in the dynamics of fields in curved spacetimes. In that case, the prefactor $\exp(2\alpha\beta t)$ corresponds to the volume density factor $\sqrt{-g}$ of spacetime. The other way to get friction is by

[1] The action (S4) is defined up to an overall normalization constant and we can choose such a constant so that the constant acceleration term in (S3) is 1. In other words, if $\lambda \neq 0$ we can rescale $S_{\rm RW} \to -mS_{\rm RW}$, where $m := -(\lambda\alpha^2\beta^2)^{-1}$ is something we might call the "conditioning mass." This is an intuitive measure of the inertia the subject experiences during learning. When the magnitude or salience of the US or the salience of the CS decrease, the mass $|m|$ increases and the less efficient the conditioning will be. The heavier the mass, the longer the learning will take to reach a certain strength of association. Note that we have chosen the arbitrary rescaling of the action in such a way that the conditioning mass depends on all the parameters we would expect to affect the inertia of learning on empirical grounds. But for practical purposes, one can work with the original action (S4).

the conventional non-variational approach to classical mechanics, where the Lagrangian does not depend explicitly on time. By definition in this case, the equation of motion is not (S6) but

$$\frac{d}{dt}\frac{\partial\mathcal{L}}{\partial\dot{v}}-\frac{\partial\mathcal{L}}{\partial v}=Q\,,\qquad Q=-2\alpha\beta\dot{v}\,, \tag{S7}$$

where Q is the friction force. The final result is the same, Eq. (S3). A third way to obtain the RW model, without friction but with an inverted potential, can be found in section 6.1 *Quantum RW model*.

The Lagrangian (S5a) has a clear interpretation in physics: it describes the one-dimensional dynamics of a nonrelativistic particle with positive mass and in a potential $U(v)$. The particle, or worldline $v(t)$, is nonrelativistic because of the form of the kinetic term $m\dot{v}^2/2$. It has a positive mass because, in our case, $m=+1$. The potential $U(v)$ (**Figure 4** in the paper) has a quadratic part, a linear part and a constant term, but it is just quadratic when expressed in terms of the variable

$$x:=\lambda-v\,. \tag{S8}$$

This is the potential of a *damped harmonic oscillator*, i.e., a particle with trajectory $x(t)$ attached to a spring with positive spring constant $k=(\alpha\beta)^2$ and subject to friction. The classical equation of motion (S3) is rewritten as

$$\ddot{x}+2\Omega\dot{x}+\Omega^2x=0\,,\qquad \Omega:=\alpha\beta\,, \tag{S9}$$

where we introduced a “frequency” Ω proportional to the salience of the US and of the CS. In excitatory conditioning, the particle rolls down the potential well from the point $x=\lambda$ ($v=0$) to the global minimum at $x=0$ ($v=\lambda$), where it stops due to the finely tuned friction with damping coefficient $2\alpha\beta$ (**Figure 4** in the paper). In extinction, the minimum is shifted to the origin but the particle behavior is similar, rolling from $v=\lambda$ down to $v=0$.

The potential (S5b) in the Lagrangian (S5a) gives rise to the equation of motion (S9), which is very special: the damping coefficient 2Ω is exactly twice the frequency of the oscillator. In general, this would be considered as a fine tuning of the parameters of the model because the friction term makes the particle stop precisely at the bottom of the potential. If we relax this condition but still allow for friction, the most natural outcome will be that the particle will start to oscillate around the minimum of the potential, eventually sitting on top of it when its kinetic energy is exhausted. This is achieved simply by changing Eq. (S5b) as

$$U(v)=\frac{(\alpha\beta)^2+\mu^2}{2}(v-\lambda)^2 \tag{S10}$$

where μ is a constant. Equation (S3) is then replaced by

$$\ddot{v}+2\Omega\dot{v}+(\Omega^2+\mu^2)(v-\lambda)=0\,,\qquad \Omega=\alpha\beta\,, \tag{S11}$$

whose solution is Eq. (8) of the main paper. This profile has concrete realizations in electronic engineering and signal processing and is called “step response” in that context (the oscillatory pattern is called “ringing”). When $\mu\neq 0$, the particle is subject to a friction force Q given by plugging Eq. (8) of the main paper into Eq. (S7).

3.2 Classical dynamics of the Rescorla–Wagner model

For future explorations, we note that it is possible to recast any model of Pavlovian conditioning as a dynamical system described by the action

$$S=\int_0^T dt\,\left[\sum_{i=1}^N\frac{\dot{v}_i^2}{2}-\sum_{i=1}^N U_i(v_i)-W(v_1,\ldots,v_N)\right]\,, \tag{S12}$$

where the functions $v_i=v_i(t)$ are the association strengths related to N conditioned stimuli (N “particles”), U_i is the potential energy of each particle, and the term $W(v_1,\ldots,v_N)$ is an interaction describing how the particles affect one another by cross-terms. The total potential energy of the system is $\sum_i U_i+W$.

Having already discussed the RW model for a single CS (Hull, 1943), we now consider the RW model with many stimuli (Rescorla and Wagner, 1972; Wagner and Rescorla, 1972), the Mackintosh attentional model (Mackintosh, 1975b), and a nonlinear approximation to Mackintosh model recently proposed by one of the authors (Calcagni, 2018). Notice that, in some cases, it may be more convenient to recast the system with different variables (if available) than the association strength in (S12).

The Rescorla–Wagner model (Rescorla and Wagner, 1972; Wagner and Rescorla, 1972) is an extension of the single-cue model to many conditioned stimuli. The trial-by-trial change in each strength of association $v_n^{(i)}$, $i = 1, \dots, N$, is

$$\Delta v_n^{(i)} = \alpha_i \beta \left[\lambda - \sum_{j=1}^{N} v_{n-1}^{(i)} \right] , \tag{S13}$$

which, in the continuum limit, translates to the differential equations

$$\dot{v}_i = \alpha_i \beta \left(\lambda - \sum_{j=1}^{N} v_j \right) , \qquad i = 1, \dots, N , \tag{S14}$$

$$\ddot{v}_i = \alpha_i \left(\sum_{j=1}^{N} \alpha_j \right) \beta^2 \left(\sum_{k=1}^{N} v_k - \lambda \right) . \tag{S15}$$

The latter stems from the multiparticle action (S12) with

$$U_i(v_i) = -\frac{1}{2} \alpha_i \left(\sum_j \alpha_j \right) \beta^2 (v_i - \lambda)^2 , \tag{S16}$$

$$\begin{aligned} W(v_1, \dots, v_N) &= -\left(\sum_j \alpha_j \right) \beta^2 \left(\sum_i \alpha_i v_i \right) \sum_{k \neq i} v_k \\ &= -\left(\sum_j \alpha_j \right) \beta^2 \sum_i \sum_{k \neq i} (\alpha_i + \alpha_k) v_i v_k , \end{aligned} \tag{S17}$$

as one can check from Eq. (S6) for each v_i. In physics, this would correspond to a system of interacting, inverted harmonic oscillators (see section 6.1 *Quantum RW model* for the single inverted oscillator). It is easy to check that the energy of each inverted oscillator is not conserved individually due to interactions.

3.3 Classical dynamics of the Mackintosh model and its nonlinear approximation

For one CS, the attentional model by Mackintosh is described by

$$\Delta v_n = \alpha_{n-1} \beta (\lambda - v_{n-1}) , \qquad \Delta \alpha_n = \gamma (\lambda - v_{n-1}) . \tag{S18}$$

In the continuum,

$$\dot{v} = \beta \alpha (\lambda - v) , \qquad \dot{\alpha} = \gamma (\lambda - v) , \tag{S19}$$

which can be combined as $\partial_t [v - \beta \alpha^2 / (2\gamma)] = 0$. Therefore, v and α are related by

$$v(t) = \frac{\beta}{2\gamma} \alpha^2(t) + \lambda - c , \tag{S20}$$

where c is a constant. Plugging this back into (S19), one obtains two first-order equations decoupled in v and α, with unique solutions

$$v(t) = \lambda - c\,\mathrm{sech}^2\left(\sqrt{\frac{\gamma\beta c}{2}}\,t\right), \tag{S21a}$$

$$\alpha(t) = \sqrt{\frac{2\gamma c}{\beta}}\tanh\left(\sqrt{\frac{\gamma\beta c}{2}}\,t\right). \tag{S21b}$$

Since $v(0) = \lambda - c$ and, for $c > 0$, $v(\pm\infty) = \lambda$, excitatory conditioning is obtained for $c = \lambda$. Comparing with Eq. (3) of the main paper, one immediately sees that the learning curve of the continuous Mackintosh model is less steep at the beginning of the conditioning, a phenomenon we dubbed (we will presently see why) "uphill learning" in Calcagni (2018). Mackintosh's model for two or more cues corresponds to a multi-particle system we will not write down here.

The construction of a Lagrangian is somewhat problematic for this model. In fact, *a priori* the strength of association v and the salience α of the CS are independent variables and one should consider a Lagrangian $\mathcal{L}[v,\alpha] = \dot{v}^2/2 + \dot{\alpha}^2/2 - U_1(v) - U_2(\alpha) - W(v,\alpha)$ which is varied with respect to the two degrees of freedom v and α. However, there is no such action. From (S19), we have

$$\ddot{v} = -\beta^2\alpha^2(\lambda - v) + \gamma\beta(\lambda - v)^2, \qquad \ddot{a} = \gamma\beta\alpha(\lambda - v). \tag{S22}$$

If we ask to vary $\mathcal{L}$ with respect to v and α independently using the Euler–Lagrange equations (S6), then the first equation in (S22) suggests an interaction term $W = \beta^2\alpha^2(\lambda v - v^2/2) + d$ for some constant d, while the second equation in (S22) requires $W = \gamma\beta\alpha^2 v/2$. Even setting $d = 0$, these two expressions can never match because the first one has an extra $O(\alpha^2 v^2)$ term. Therefore, if we insist in describing the model with a dynamical action, we must first use the relation (S20) to recast Eq. (S22) into a single nonlinear differential equation. Let us do this first for the association strength v. Equation (S21a) can be recast as

$$\dot{v} = \pm\sqrt{-2U(v)}, \tag{S23}$$

$$\begin{aligned} U(v) &= -\gamma\beta(v - \lambda + c)(v - \lambda)^2 \\ &= -\gamma\beta[(c-\lambda)\lambda^2 + (3\lambda - 2c)\lambda v - (3\lambda - c)v^2 + v^3]. \end{aligned} \tag{S24}$$

Notice that the writing (S23) is equivalent to $\ddot{v} + U'(v) = 0$. Then, the Lagrangian reads

$$\mathcal{L}_{\mathrm{Mac}} = \frac{\dot{v}^2}{2} - U(v), \tag{S25}$$

while the energy is

$$E_{\mathrm{Mac}} = \gamma\beta(v - \lambda - c)(\lambda - v)^2 + U(v) = 0. \tag{S26}$$

The potential (S24) is understood as valid in the interval $0 \leqslant v \leqslant \lambda$, while outside this interval one should impose the infinite barriers as in (S5).

Mathematically, the only but important difference with respect to the RW model is in the form of the potential: a polynomial of order 2 in the RW case and of order 3 in the Mackintosh case, with the sign of the linear and quadratic terms flipped; see **Figure S12**. While in the RW model excitatory conditioning is represented by a particle rolling up the potential towards the absolute maximum, in the Mackintosh model the particle has first to roll down a trough (the local minimum in the figure) before climbing up the local maximum. Defining the action with an overall extra $-$ sign, this motion consists in climbing up a hill before rolling down the local minimum; this is the dynamical description of "uphill" learning.

It may be more convenient to recast the system in terms of the variable α. The problem of the potential in (S25) is that it is unbounded from below. The solution (S21a) is of "rolling" type, i.e., the particle moves

from some initial position down to the local minimum or up to the local maximum. In order to avoid falling indefinitely down or climbing upwards the potential, we have imposed the initial conditions typical of a learning process. However, these initial conditions are rather unnatural inasmuch as they do not correspond to a local extremum of the potential: so to speak, the particle starts with non-zero velocity at a slope of the potential. This configuration is not suitable to describe quantum solutions of the quantum system, which should always begin from or end at a static configuration.

Such is the characteristic of the solution (S21b). The second expression in (S19) can be written as

$$\dot{\alpha} = \pm\sqrt{-2U(\alpha)}\,, \tag{S27}$$

$$U(\alpha) = -\frac{1}{2}\left[\gamma c - \frac{\beta}{2}\alpha^2\right]^2, \tag{S28}$$

corresponding to the Lagrangian

$$\tilde{\mathcal{L}}_{\rm Mac} = \frac{\dot{\alpha}^2}{2} - U(\alpha)\,. \tag{S29}$$

The potential $U(\alpha) \leqslant 0$ is quartic and is bounded from above (**Figure S13**). For $c > 0$, its two maxima are at $\alpha = \pm\sqrt{2\gamma c/\beta}$. In particular, to reach the asymptotic value $\alpha = 1$ it must be $\gamma = \beta/(2c)$. The solution (S21b) is a *kink* interpolating between these maxima. In the present case, however, $t \geqslant 0$ and the solution runs from the local minimum at $\alpha = 0$ to the positive maximum. This corresponds, in particular, to the excitatory case $c = \lambda$.

Let us pause for a moment and discuss a caveat. To get an extinction curve, one should have $v(0) = \lambda$ as initial condition and $v(\pm\infty) = 0$. Then, setting $\lambda = 0$ one would have to impose $c < 0$; for this range of values, v and α remain real valued but become periodic, $v \propto \cos^{-2}$ and $\alpha \propto \tan$. This behavior does not correspond to extinction. Another possibility is to consider the branch $t \in (-\infty, 0]$ that we ignored in all the other models. Since v is even in time, following $v(t)$ from the infinite past until $t = 0$ corresponds to decrease the association strength from λ to 0. However, this is a solution of the equations of motion with a nonvanishing parameter λ, which means that the US is offered at the end of each trial. Clearly, this does not correspond to a realistic extinction experiment. Rather, it is a sort of "rewinding" of an excitatory experiment backwards in time or, conversely, an excitatory process with a different parametrization of time (t running from 0 to $-\infty$). The latter interpretation can also be reached by taking the Mackintosh proposal as an approximate model and assuming α to sit at the maximum at $\alpha = -\sqrt{2\gamma c/\beta}$. In that case, for $c = \lambda$ we would have a RW model with $t \to -t$ and the negative branch would be physically the same as the positive branch.

We conclude this section with a side remark. The system of differential equations (S19) could be solved exactly, while the treatment of the discrete system (S18) is more complicated. This motivated one of the authors to propose a nonlinear model approximating Mackintosh's when the parameter γ is very small (Calcagni, 2018). Given two parameters $0 < \alpha_{\rm min} < \alpha_{\rm max} \leqslant 1$ representing the minimum and maximum value of the salience of the CS, this model is

$$\alpha_n = \alpha_{\rm min} + (\alpha_{\rm max} - \alpha_{\rm min})\frac{v_n}{\tilde{\lambda}}\,, \tag{S30}$$

$$\begin{aligned}\Delta v_n &= \alpha_{n-1}\beta(\lambda - v_{n-1})\\ &= \beta\left[\alpha_{\rm min} + (\alpha_{\rm max} - \alpha_{\rm min})\frac{v_{n-1}}{\tilde{\lambda}}\right](\lambda - v_{n-1})\\ &= A - Bv_{n-1} - Cv_{n-1}^2\,,\end{aligned} \tag{S31}$$

where $A = \beta\alpha_{\rm min}\lambda$, $B = \beta(\alpha_{\rm min} - C\lambda)$, $C = (\alpha_{\rm max} - \alpha_{\rm min})/\tilde{\lambda}$. In the continuum, we have $\dot{v} = A - Bv - Cv^2$, i.e.,

$$\ddot{v} = -AB + (B^2 - 2AC)v + 3BCv^2 + 2C^2v^3\,. \tag{S32}$$

The profile $U(v)$ is the same as in **Figure 4** of the paper when $\gamma = O(10^{-2})$, while for larger γ the local maximum is lowered (**Figure S14**).

If we compare Eqs. (S32) and (S23), we immediately see that this nonlinear model collapses to Mackintosh's model when the cubic term is negligible, $C \ll 1$, which happens if $\alpha_{\rm max} \simeq \alpha_{\rm min} \simeq \alpha$. All the coefficients can be mapped easily into one another. For instance, the $O(v^2)$ terms in Eqs. (S32) and (S23) match if $C \simeq \gamma/\alpha$.

4 STOCHASTIC MODEL AND SPECTRAL ANALYSIS

4.1 Stochastic model

Consider the original equation (1) of the main paper for the change in associative strength from one trial to the next: $\Delta v_n = \alpha\beta(\lambda - v_{n-1})$, which we can also write as $v_n = (1-\alpha\beta)v_{n-1} + \lambda\alpha\beta$. Now we promote v_n to a random variable $V_n = v_n + \xi_n$, where v_n and ξ_n are, respectively, the deterministic and stochastic parts. This is tantamount to consider the stochastic version of the previous equation, with an added random noise source η_n:

$$V_n = (1-\alpha\beta)V_{n-1} + \lambda\alpha\beta + \eta_n\,, \tag{S33}$$

where $\eta_n = \xi_n - (1-\alpha\beta)\xi_{n-1}$. This is called a first-order autoregressive process (ARP) with drift $\lambda\alpha\beta$ and colored noise η_n. When η_n has a normal distribution (white noise) and in the limit $\alpha\beta \to 0$, this reduces to a random walk (Brownian motion). In the case of Pavlovian conditioning, it would correspond to a baseline random behavior in the presence of non-salient stimuli.

Assume that a completely noise-free learning curve were described by the RW model (S2). This curve is smooth at all times and this should reflect in its frequency decomposition. To see this, we take its cosine *Fourier transform*:

$$\begin{aligned}\tilde v(\omega) &= \mathcal{F}[v(t)] := 2\int_0^{+\infty} dt\,\cos(\omega t)\,v(t)\\ &= -\frac{2\lambda\alpha\beta}{(\alpha\beta)^2+\omega^2} + 2\pi\lambda\delta(\omega)\,,\end{aligned} \tag{S34}$$

where ω is the frequency and δ is the Dirac delta distribution (identically equal to zero for $\omega \neq 0$ and to ∞ at $\omega = 0$). We employ the cosine Fourier transform, typically used in signal processing, because we want a real-valued results and $v(t)$ has support only in $t \geqslant 0$. Without loss of information, we can remove the zero mode $\omega = 0$ and restrict the frequency range to $\omega > 0$ ($\tilde v(-\omega) = \tilde v(\omega)$ is even in ω), so that the delta term drops out. The *power spectral density* is then defined as the modulus square of the Fourier transform of the signal:

$$S(\omega) := |\tilde v(\omega)|^2 = \frac{4(\lambda\alpha\beta)^2}{[(\alpha\beta)^2+\omega^2]^2}\,. \tag{S35}$$

This profile is depicted in **Figure S15** in a log-log scale. Short frequencies have a constant power up to some point $\omega_0 = (4\lambda\alpha\beta)^{-2}$,[2] where the spectral density drops steadily to zero. This means that the profile $v(t)$ is mainly made of frequencies $\omega \lesssim \omega_0$, while frequencies $\omega \gg \omega_0$ are not important. These large frequencies correspond to small-scale details of $v(t)$ but, since $v(t)$ is completely smooth, its small-scale structure is empty: zooming into it does not give more information. This signal is further distorted by the limited size of the data sample, by small but smooth ripples. Thus, if data follow a smooth learning curve different from RW, then a spectral analysis should find an $S(\omega)$ different from that shown in **Figure S15**. By itself, this should draw our interest in determining the power spectral density of data.

However, the spectral analysis can uncover much more. In general, data are noisy. This noise can come from statistical error or from intrinsic sources. Statistical error has a Gaussian distribution and is therefore *white noise*. White noise is a random signal with a uniform probability distribution of frequencies (**Figure S16**). In general, random signals called *colored noise* can be characterized by the power spectral density

[2] The first derivative of (S35) is proportional to ω/ω_0 and is therefore small (i.e., $S(\omega)$ is approximately constant) when $\omega/\omega_0 \ll 1$.

(up to an overall constant)

$$S_a(\omega) = \frac{1}{\omega^a}\,, \tag{S36}$$

where a is a constant exponent (usually denoted as α in the literature; to avoid confusion with the CS salience, we change notation here). White noise corresponds to $a = 0$, while other popular cases are *pink noise* ($a = 1$, S_1 decreases linearly with the frequency), *Brownian noise* ($a = 2$, S_2 decreases quadratically), *blue noise* ($a = -1$, S_{-1} increases linearly with the frequency), and so on (**Figure S16**). The spectrum (S36) is better visualized in log-log plot, since in that case the parameter a is nothing by the slope of the line $\log S_a = -a \log \omega$.

As we said above, experimental data of associative learning processes are usually noisy at small time scales, i.e., the trial-by-trial response variation is usually greater than any long-term variation around the asymptote of learning. Therefore, the spectral density of a realistic learning curve following the RW model should be of the form of **Figure S15** with a noise signal superposed and dominating those frequencies where $S(\omega)$ drops. In other words, the total signal $S_{\rm tot}(\omega) = S(\omega) + S_a(\omega)$ is approximately equal to $S_{\rm tot}(\omega) \simeq S(\omega)$ (Eq. (S35)) at small frequencies $\omega \lesssim \omega_0$, while $S_{\rm tot}(\omega) \simeq S_a(\omega)$ at large frequencies $\omega \gg \omega_0$. Simulated signals of this kind are shown in **Figure S17**. Here we can also appreciate the distortion (smooth ripples) coming from the limited size of the data sample.

4.2 Spectral analysis

Figure S18 (most most normative subject at the session-by-session time scale) and **Table S2** (RW best-fit values for all subjects) are helpful to compare trial-by-trial with session-by-session data. In particular, data do not become smoother when changing the scale and there is greater dispersion than in binned data (session-by-session, see **Table S1**).

For the nonlinear fit of our data, we used a sampling of frequencies of $\Delta\omega = 0.001$, but we checked that a sampling ten times coarser does not change the results significantly. Also, if one fits up to a smaller maximum, e.g., $\omega = 5$ or $\omega = 10$, one does obtain a nonvanishing a for several subjects. However, this may be due to considering a region of frequencies where the negative slope of the background profile in **Figure S15** still dominates over the noise signal.

We collect the spectra of all the subjects of our experiment. Due to failure of recordings, the total number of points of subjects 1-5, 1-6, and 1-7 is smaller than the maximum 3960: respectively, 3828, 3894, and 3916. This causes no problem since we have plenty of statistics. The exponential-dominated smooth region (plateaux near $\omega = 0$) in **Figure S17** is not visible in these figures because they span a larger frequency range; however, one can check that the spectra at low frequencies are of the same form as the simulated white-noise spectrum in **Figure S17**.

Fitting the power spectra from $\omega = 0.1$ to $\omega = 30$, we get the results of **Tables S3**, **S4**, already commented in the main paper.

Note that the spectral analysis of the noise signal occurs in a frequency region unaffected by whether the background model is RW or the oscillatory one. The power spectral density of the latter would only differ in the position of the right end of the plateau in **Figure S15** and in an extra bump just at the onset of the slope. When fitting high-frequency data, these details are subdominant with respect to the main noise trend and, in fact, they do not appear in **Figures S19 S20**.

5 OTHER MODELS OF FLUCTUATING RESPONSE

5.1 Fractional stochastic model

We want to explore the possibility of a long-range effect in data or, more precisely, whether the time series of responses has long-memory correlations. The previous model accounts for local fluctuations in the response but does not contemplate the chance that, for whatever reason, the learning history of the subjects can affect future response more heavily than predicted by the RW model, in either its deterministic or stochastic version. This situation is described, for instance, by an autoregressive fractionally integrated moving average (ARFIMA) process. Without entering into many details, we can model this process as follows. In the continuous-time limit, the AR process with drift and colored noise (S33) takes the form

$\dot{V}(t) = \alpha\beta[\lambda - V(t)] + \eta(t)$. A random ARFIMA-like process with long memory would be described by

$$\partial_t^\gamma V(t) = \alpha\beta[\lambda - V(t)] + \eta(t)\,, \tag{S37}$$

where ∂_t^γ is a fractional derivative of order γ (Kilbas et al., 2006). There are many definitions of fractional derivative; here we will take the left Caputo derivative $(\partial^\gamma f)(t) := [\Gamma(1-\gamma)]^{-1}\int_0^t dt'(t-t')^{-\gamma}\partial_{t'}f(t')$, where Γ is Euler function and $0 < \gamma < 1$. Fractional derivatives are commonly employed in statistical, financial, or physical systems with memory.

In this descriptive model, the biggest impact on behavior is given by the fractional derivative rather than the noise term. For this reason, we can ignore η and consider the deterministic equation

$$\partial_t^\gamma v = \alpha\beta(\lambda - v)\,, \tag{S38}$$

whose solution is the Mittag-Leffler function (Haubold et al., 2011):

$$v(t) = \lambda[1 - E_\gamma(-\alpha\beta t^\gamma)]\,. \tag{S39}$$

When $\gamma = 1$, $E_1(-\alpha\beta t) = \exp(-\alpha\beta t)$ and one recovers (S2). The learning curve (S39) is shown in **Figure S21**. Compared with the RW curve, (S39) is steeper at early times and increases much slower at late times.

Comparing the BIC and AIC of the best fit of normalized session-by-session individual data with the BIC and AIC of the RW model and the DOM shown in **Table 2** of the paper, one can check that the fractional model is unfavored by all subjects except 1-4, which is a false positive: $\gamma = 1$ within the error uncertainty. In fact, all fits select $\gamma = 1$ as the value of the fractional exponent. Since the deterministic model (S38) does not work, we can also exclude the stochastic version (S37), which exhibits the same global trend.

5.2 Random-fractal model

Calcagni (2018) noted that, instead of a learning curve, the set of points of the association strength in a Pavlovian conditioning process can be described by a fractal, a somewhat peculiar set defined recursively whose points can be totally disconnected from one another and whose dimension can be noninteger. A learning process is then represented as a "hopping" on a fractal, each trial or session corresponding to an iteration level of the set. This reinterpretation, applied to the RW, Mackintosh, and Pearce–Hall models, does not change the prediction of any of these deterministic (i.e., not involving probabilities) learning models, but it offers a geometric way to characterize not the efficiency of a training program *per se*, but for a given subject.

For instance, a so-called Cantor set is associated with the RW model and its dimension (more precisely, Hausdorff dimension) is $d_{\rm H}(\mathcal{C}) = d_{\rm C}(\mathcal{C}) = -\ln 2/\ln(1-\alpha\beta)$. We can calculate this quantity from the individual session-by-session fits of **Table S1**. There is great variability in the dimension. 1-1: $d_{\rm H} \approx 2.4$, 1-2: $d_{\rm H} \approx 13.1$, 1-3: $d_{\rm H} \approx 6.1$, 1-4: $d_{\rm H} \approx 0.6$, 1-5: $d_{\rm H} \approx 7.5$, 1-6: $d_{\rm H} \approx 7.5$, 1-7: $d_{\rm H} \approx 2.9$; 2-1: $d_{\rm H} \approx 2.8$, 2-2: $d_{\rm H} \approx 4.0$, 2-3: $d_{\rm H} \approx 2.2$, 2-4: $d_{\rm H} \approx 1.9$, 2-5: $d_{\rm H} \approx 0.5$, 2-6: $d_{\rm H} \approx 19.6$, 2-7: $d_{\rm H} \approx 11.7$, 2-8: $d_{\rm H} \approx 2.7$. The large the saliences $\alpha\beta$, the smaller the dimension (the fractal set becomes a collection of disconnected sparse points) and the more efficient the conditioning, i.e., fewer the sessions needed to reach the asymptote of learning. Such is the case of subjects 1-4 and 2-5, which maintained a steady response already in the first 10 sessions. On the other hand, the larger the dimension the longer it took to reach the asymptote, as was the case of subjects 1-2, 2-6, and 2-7, all of which showed a slow steady increase almost until the end of the experiment.

This description, alternative to the usual one, may be interesting by itself, but the fractal interpretation does more than that when a random element is introduced. The deterministic model can be extended to a probabilistic one when the saliences $\alpha\beta$ take a random value in a given distribution with support between 0 and 1. This can happen in different situations, from a controlled experimental design of partial reinforcement with randomized schedule (the CS or the US can be either present or absent at any given trial) to the natural environment of the subject with everchanging stimuli. Or, according to the hypotheses put forward in the $1/f$ cognitive literature (section *Colored stochastic model of individual behavior – Theory*), random variations of $\alpha\beta$ may happen due to the internal flickering of the subject's cognitive modules.

In all cases, because of the characteristics of the geometric interpretation, one expects these random fluctuations to be relatively *small* with respect to the absolute asymptote of learning and progressively

damped (Calcagni, 2018). Neither feature is seen in the data: behavioral fluctuations are not damped in time can be as large as $O(\lambda)$ and go well above or below the theoretical asymptote found with the best fit. This finding does not rule out the fractal picture because, as we said, it is a repackaging of the RW model in terms of a geometric language. However, it does limit the scope of application of random fractals, at least in experimental designs like this where the US is not presented on a deterministic schedule: random variations of $\alpha\beta$ do not describe observed and observable response instabilities but, rather, some subdominant and perhaps undetectable effect.

6 QUANTUM RW MODEL

6.1 Motivation

Having described, in the main paper, several well-known models of Pavlovian conditioning in terms of the classical mechanics of pointwise particles, we make a new step: we quantize them. In physics, "quantization" is a mathematical procedure that uses a classical system as the basis to construct a physically different system governed by the law of quantum mechanics. These laws are still governed by the least-action principle, they describe the behavior of microscopic objects and recover the classical system in a certain limit. In the present context of behavioral psychology, we want to quantize the learning system not just "because we can," but because we want to check whether the dynamical paradigm can also explain quantitatively the response fluctuations observed in experiments. This question is interesting in its own right, independently of whether the quantum model explains data successfully or not.

The tenet of this model is that response variations can be described by a different type of randomness (which we call "quantum" only because we use the same mathematics of quantum mechanics) than that of an aleatory noise as assumed in section 4.1. Of course, we do not imply that the learning process is quantum in any physical sense.

A classical pointwise particle moving along a smooth trajectory $x(t)$ becomes, upon quantization, a wave-function $\Psi(x,t)$ which indicates the probability to find the particle at position x at time t. It is not our purpose to introduce the reader to the main notions of quantum mechanics. What matters here is to understand what happens if we quantize conditioning models: do these models predict phenomena in animal behavior not contemplated by standard learning? The answer is in the affirmative.

In "classical" learning, the response is predicted deterministically by the learning curve, so that at time t after the onset of training the CS-US association strength will exactly be $v(t)$; this association strength is then identified with the amount of response of the subject, for instance, the number of licks. Experimental uncertainty forbids to take this picture too literally, and exact predictions must be checked against data with error bars. However, in "quantum" learning the prediction itself is not deterministic and we cannot claim that at time t the subject will respond with strength v. Instead, we can only give a probability distribution $\mathcal{P}(v,t)$ saying what the chance is that at time t the subject will respond with strength v. This uncertainty is *not* due to experimental errors and it will be there even in the most unrealistic case of an error-free observation. In other words, it is intrinsic to the behavior of the animal. Whether this feature is of motor or cognitive origin is a problem one might want to understand after checking the theory experimentally. What matters here is that we can predict the exact shape of the probability distribution $\mathcal{P}(v,t)$ if we follow the quantization procedure so successful in physics. The point-particle trajectory $v(t)$ is replaced by a wave-function $\Psi(v,t)$, solution to the quantum dynamics, that can spread throughout the whole domain $0 < v < \lambda$. The probability to have a certain association strength v at time t is given by $\mathcal{P}(v,t) = |\Psi(v,t)|^2$. The characteristic of these *quantum* models of Pavlovian conditioning is that they have no way to maintain perfect conditioning (i.e., $v = \lambda$) indefinitely. After some sojourn time determined by the salience of the stimuli, the subject can exhibit a nonoptimal or even overoptimal association strength away from perfect conditioning, even if the CS-US pair is presented in each trial. We will describe these effects with the simplest available example: the quantum RW model.

6.2 Construction of the quantum RW model

6.2.1 Preliminaries: classical setting

Since the friction term in (S3) can introduce unnecessary complications, we first recast the classical model as an *inverted harmonic oscillator*. Taking the time derivative of $\dot{v} = \alpha\beta(\lambda - v)$ (Eq. (2) in the main

paper) but replacing $\dot{v}$ with its explicit expression, we obtain the equation of motion

$$\ddot{v} - (\alpha\beta)^2 v + \lambda(\alpha\beta)^2 = 0\,, \tag{S40}$$

which can be derived from the action

$$S_{\rm RW} = \int_0^T dt\, \mathcal{L}_{\rm RW-inv}\,, \tag{S41}$$

where

$$\mathcal{L}_{\rm RW-inv} = \frac{\dot{v}^2}{2} - U(v)\,, \tag{S42a}$$

$$U(v) = \begin{cases} -\frac{(\alpha\beta)^2}{2}(v-\lambda)^2 & (0 \leqslant v \leqslant \lambda) \\ +\infty & (v < 0 \quad \text{or} \quad v > \lambda) \end{cases}\,. \tag{S42b}$$

In excitatory conditioning, the particle rolls up its hill-top potential from the global maximum $v = 0$ to the point $v = \lambda$ at which we placed an infinite potential barrier. This barrier is simply a way to force the dynamics into the interval $0 \leqslant v \leqslant \lambda$.

Applying the variational principle, the Euler–Lagrange equations (S6) yield (S40). This time, the particle is attached to a spring with negative spring constant $k = -(\alpha\beta)^2 < 0$. In terms of the variable (S8),

$$\ddot{x} - \Omega^2 x = 0\,, \qquad \Omega = \alpha\beta\,. \tag{S43}$$

The energy of the particle is a constant of motion and it vanishes. Since Eq. (2) in the main paper is equivalent to $\dot{v} = \sqrt{-2U(v)}$,

$$E_{\rm RW} := \frac{\dot{v}^2}{2} + U(v) = 0\,. \tag{S44}$$

Therefore, no matter what type of conditioning process enforced, the initial and final state will have equal energy. The energy found in (S44) vanishes but one can always shift the potential by a constant that does not appear in the equation of motion, so that E can take positive or negative values. For the particle to climb the potential up to the maximum, the initial kinetic energy $E_{\rm kin} = [\dot{v}(0)]^2/2$ must be positive in order to cancel the negative contribution $U(0) = -(\alpha\beta\lambda)^2/2$ of the potential. At the final point, $E_{\rm kin} = 0 = U$. Thus, comparing with the friction model, the hill-top model has the disadvantage of needing a potential barrier and requiring a somewhat unnatural initial condition (the particle starts exactly at the point on the slope which ensure it reaches the hill-top asymptotically), but on the other hand it is a conservative system.

In preparation for the quantum analysis, we recast the RW model in Hamiltonian formalism. Defining the momentum

$$p := \frac{\delta S_{\rm RW}}{\delta \dot{x}} = \dot{x}\,, \tag{S45}$$

the classical *Hamiltonian* $H := p\dot{x} - \mathcal{L}$ from (S5) is

$$H = \frac{1}{2}(p^2 - \Omega^2 x^2)\,. \tag{S46}$$

This expression is valid within the well $0 \leqslant x \leqslant \lambda$. The variables p and $x = \lambda - v$ are said to be canonically conjugate and their Poisson bracket $\{A, B\} := (\partial A/\partial x)(\partial B/\partial p) - (\partial A/\partial p)(\partial B/\partial x)$ is

$$\{x, p\} = 1\,. \tag{S47}$$

The general classical solution of the equation of motion (S9) is

$$x(t) = x_0 \cosh(\Omega t) + \frac{p_0}{w}\sinh(\Omega t)\,, \tag{S48}$$

where $x_0 = x(0)$ and $p_0 = p(0)$ and the hyperbolic functions are defined by $\cosh z := (e^z + e^{-z})/2$ and $\sinh z := (e^z - e^{-z})/2$. The energy is $E_{\rm RW} = H[x(t), p(t)] = (p_0^2 - \Omega^2 x_0^2)/2$.

6.2.2 Quantization

As we just saw, the usual RW model is nothing but the classical inverted harmonic oscillator. The quantum inverted oscillator is also well known because it is one of the few cases that admit a fully analytic treatment (Barton, 1986; Kemble, 1935; Shimbori, 2000; Shimbori and Kobayashi, 2000; Yuce et al., 2006). We will rely mainly on the results of Barton (1986), which we will review first and then interpret them in the fresh context of learning by conditioning. The starting point is the classical Hamiltonian formalism given by the Hamiltonian (S46) and the Poisson bracket (S47) for the canonical variables x and p. We promote the latter to operators $\hat{x}$ and $\hat{p}$ acting on a Hilbert space whose states $\Psi(x,t)$, also denoted by $\langle x|\Psi\rangle$, are called wave-functions. The position operator $\hat{x}$ in the Schrödinger picture can be chosen to act multiplicatively and the momentum operator $\hat{p}$ as a first-order partial differential derivative (this choice is called position representation):

$$x \to \hat{x} := x\,, \qquad p \to \hat{p} := -i\bar{h}\frac{\partial}{\partial x}\,, \tag{S49}$$

where $\bar{h}$ is a constant. In physics, this would be Planck constant $\hbar$ ("h bar") but in psychology we do not know yet its magnitude and we call it with another symbol ($\bar{h}$, "bar h"). The Poisson bracket (S47) is converted into the commutator

$$\{\,\cdot\,,\,\cdot\,\} \to \frac{1}{i\bar{h}}[\,\cdot\,,\,\cdot\,]\,, \tag{S50}$$

where $[\hat{A},\hat{B}] := \hat{A}\hat{B} - \hat{B}\hat{A}$. Consequently,

$$[\hat{x},\hat{p}] = i\bar{h}\,. \tag{S51}$$

Denoting by $\langle\hat{A}\rangle = \langle\Psi|\hat{A}|\Psi\rangle$ the expectation value of an operator $\hat{A}$ on a state $|\Psi\rangle$, one defines the standard deviation $\Delta A = \sqrt{\langle\hat{A}^2\rangle - \langle\hat{A}\rangle^2}$. Then, for any quantum state and any two noncommuting canonically conjugate operators $\hat{A}$ and $\hat{B}$ it is easy to prove Heisenberg uncertainty principle

$$\Delta A\,\Delta B \geqslant \frac{\bar{h}}{2}\,, \tag{S52}$$

stating that one cannot have arbitrarily small standard deviations for both operators at the same time. In the case of the inverted harmonic oscillator and for symmetric states such that $\langle\hat{x}\rangle = 0 = \langle\hat{p}\rangle$, we have

$$\langle\hat{x}^2\rangle\langle\hat{p}^2\rangle \geqslant \frac{\bar{h}^2}{4}\,. \tag{S53}$$

If one forces the quantum particle into a state sharply peaked at a certain position x, then the expectation value of the momentum cannot be determined with arbitrary accuracy.

To determine how the wave-function $\Psi(x,t)$ evolves in time and position (association strength), we have to specify an equation for its dynamics. This is achieved in a standard way by replacing x and p in Eq. (S46) with the operators (S49), yielding the quantum Hamiltonian

$$\hat{H} = \frac{1}{2}(\hat{p}^2 - \Omega^2\hat{x}^2) = -\frac{1}{2}\left(\bar{h}^2\partial_x^2 + \Omega^2 x^2\right)\,. \tag{S54}$$

Then, the dynamics of $\Psi(x,t)$ is defined by the Schrödinger equation

$$\left(i\bar{h}\partial_t - \hat{H}\right)\Psi(x,t) = 0\,, \tag{S55}$$

with boundary conditions to be established later. A first possibility, appealing because it can allow for a probabilistic interpretation of the wave-function, is to choose boundary conditions such that the wave-function vanishes at the walls x_1 and x_2 of the potential barrier. However, it is easy to check that this configuration is not viable. Let us see why. To determine the general solution of the Schrödinger equation (S55) with potential barriers, we use the procedure of Yuce et al. (2006). Plugging the expression

$\Psi(x,t)=\exp[i\Omega x^2/(2\bar{h})-\Omega t/2]\Phi(x,t)$ into (S55) with (S54), one gets $i\dot{\Phi}+i\Omega x\partial_x\Phi+(\bar{h}/2)\partial_x^2\Phi=0$. After the variable redefinition $x=\exp(\Omega t)\,y$ and a few manipulations, the equation of motion becomes $ie^{2\Omega t}\dot{\Phi}+(\bar{h}/2)\partial_y^2\Phi=0$. Separating the variables as $\Phi(y,t)=\phi(y)\,b(t)$ with $b(t)=\exp(ie^{-2\Omega t}a/\Omega)$ and a some constant, one obtains $(\partial_y^2+4a/\bar{h})\phi(y)=0$, solved by $\phi(y)=A\sin(2\sqrt{a/\bar{h}}y)+B\cos(2\sqrt{a/\bar{h}}y)$. Overall, the general solution is

$$\begin{aligned}\Psi(x,t) &= \mathcal{N}\exp\left(i\frac{\Omega x^2}{2\bar{h}}+ie^{-2\Omega t}\frac{a}{\Omega}-\frac{\Omega t}{2}\right)\\ &\quad\times\left[A\sin\left(2\sqrt{\frac{a}{\bar{h}}}e^{-\Omega t}x\right)+B\cos\left(2\sqrt{\frac{a}{\bar{h}}}e^{-\Omega t}x\right)\right],\end{aligned}\tag{S56}$$

where $\mathcal{N}$ is a normalization constant.

For the wave-function (S56) to vanish at $x_1=0$ ($v=\lambda$ the asymptote of learning), it must be $B=0$. We also set $A=1$, since there is an overall constant $\mathcal{N}$. On the other hand, at the beginning of conditioning ($x_2=\lambda$, $v=0$) one has $\Psi(\lambda,0)=0$ only when the sine vanishes, which happens when $2\lambda\sqrt{a/\bar{h}}=n\pi$. This fixes the constant a introduced earlier:

$$a=\bar{h}\left(\frac{n\pi}{2\lambda}\right)^2,\qquad n\in\mathbb{N}.\tag{S57}$$

To fix also $\mathcal{N}$, we note that the wave-function Ψ is normalizable only if the position of one of the boundaries moves as $x_2=\lambda e^{\Omega t}$. Therefore, in order to have a probabilistic interpretation of the wave-function we assume that one of the walls is dynamical. Consequently, integrating $|\Psi|^2$ between x_1 and x_2 and imposing the result to be 1, we get

$$\int_0^{\lambda e^{\Omega t}} dx\,|\Psi(x,t)|^2=\mathcal{N}^2\frac{\lambda}{2}=1\quad\Rightarrow\quad\mathcal{N}=\sqrt{\frac{2}{\lambda}}.\tag{S58}$$

The final result for the square-integrable wave-function of the quantum inverted oscillator in a box with moving walls is

$$\begin{aligned}\Psi_n(x,t) &= \sqrt{\frac{2}{\lambda}}\exp\left[i\frac{\Omega x^2}{2\bar{h}}+ie^{-2\Omega t}\frac{\bar{h}}{\Omega}\left(\frac{n\pi}{2\lambda}\right)^2-\frac{\Omega t}{2}\right]\\ &\quad\times\sin\left(n\pi e^{-\Omega t}\frac{x}{\lambda}\right),\qquad x=\lambda-v\,.\end{aligned}\tag{S59}$$

The probability distribution of values v is

$$|\Psi_n(v,t)|^2=\frac{2}{\lambda}e^{-\Omega t}\sin^2\left(n\pi e^{-\Omega t}\frac{\lambda-v}{\lambda}\right).\tag{S60}$$

Unfortunately, the moving-wall configuration is unviable in our context. The probability density $|\Psi|^2$ is characterized by n periodic peaks in the interval $\lambda(1-e^{\Omega t})\leqslant v\leqslant\lambda$. As time increases, the peaks spread in the region $-v\gg 0$, which has no meaningful interpretation in the context of Pavlovian learning. Therefore, we must abandon the moving-wall case.

Intuitively, a wave-function vanishing at $v=0$ and $v=\lambda$ would never be able to recover the classical behavior in any limit, since it would prescribe the absence of the particle at the initial and final point of the evolution (in other words, such a wave-function would not describe a conditioning process approaching the asymptote $v=\lambda$ starting from $v=0$). From now on, we consider the case without potential barriers where the Hamiltonian is given exactly by (S54) for all $x\in\mathbb{R}$.

It is important to note that there is no quantum notion of a particle at a position x at time t. Given a square-integrable wave-function $\Psi(x,t)$ such that $\int_{-\infty}^{+\infty} dx\,|\Psi(x,t)|^2 = 1$, the density $|\Psi(x,t)|^2$ determines the probability to find the particle at a position x at time t. This probabilistic description of the particle dynamics is not due to our ignorance about the fine details of the experimental setting or of the theory: it is an intrinsic indeterminacy of Nature which has been verified in countless observations in physics. In the case of learning by conditioning models, we propose a similar setting, where the strength of association v at any given trial can be determined only probabilistically by a distribution $|\Psi(v,t)|^2$.

Since, as is well known, the Schrödinger procedure is equivalent to the path-integral quantization, one can explain these findings by interpreting "quantum" conditioning as the superposition, weighted by the classical action (S41), of all possible learning curves from $t=0$ to $t=T$. Many of these trajectories do not reach optimal learning at $t=T$ and they interfere with the classical trajectory with optimal learning producing the above-mentioned effect.

To find the most general solution of (S55), we need the *Green function* $G(x,x';t)$, which is the only solution of the Schrödinger equation that describes a point-wise source at $t=0$. It is easy to check that

$$G(x,x';t) = \sqrt{\frac{\Omega}{2\pi i\bar{h}\sinh(\Omega t)}}\exp\left\{\frac{i\Omega}{2\bar{h}\sinh(\Omega t)} \times[(x^2+{x'}^2)\cosh(\Omega t)-2xx']\right\} \tag{S61}$$

is solution of $(i\bar{h}\partial_t - \hat{H})G = 0$ with Hamiltonian (S54), such that $G(x,x';0)=\delta(x-x')$ (Barton, 1986). Then, the general solution of (S55) is given by the propagation of any wave-function $\Psi(x,0)$ in time via G:

$$\Psi(x,t) = \int_{-\infty}^{+\infty} dx'\, G(x,x';t)\,\Psi(x',0)\,. \tag{S62}$$

In fact, $(i\bar{h}\partial_t - \hat{H})\Psi(x,t) = \int dx'\,[(i\bar{h}\partial_t - \hat{H})G]\Psi(x',0) = 0$.

6.2.3 Predictions

Prediction 1: The asymptote of learning is not a durable achievement. A notable consequence of Heisenberg uncertainty principle (S53) is that equilibrium configurations cannot last forever. Suppose, for instance, that we set the initial conditions of the inverted oscillator solution (S48) such that to reproduce excitatory conditioning in the RW model: $x_0 = -p_0/\Omega = \lambda$, $x(t) = \lambda e^{-\Omega t}$. The classical system evolves from $x(0)=\lambda$ up to the maximum of the potential at $x(+\infty)=0$. Although $x=\lambda-v=0$ is reached asymptotically in time, in a real situation the subject gets close to this point in a finite time and, according to the classical theory, achieves full conditioning. This means that the subject cannot learn anything more about the association between the CS and the US: once reached the maximum of the potential, it will stay there forever. The maximum is a point of unstable equilibrium, i.e., by "perturbing the particle" a bit (in the psychological interpretation, this means to stimulate the subject with some means) it would fall down the potential again (the subject would "unlearn" the S-S association). Since the classical model has no such perturbations incorporated, this occurrence never happens in the usual RW model. However, in the quantum RW model the point of unstable equilibrium cannot be maintained due to quantum fluctuations and, after some characteristic *sojourn time* t_*, the particle is displaced away from the maximum and the subject shows signs of "unlearning" (if it falls back to the $v<\lambda$ side of the hill) or even of "overlearning" (if it falls down on the $v>\lambda$ side of the hill).

Prediction 2: There exists a quantum state such that response fluctuations do not decrease in time. Let us look for wave-functions peaked at the classical trajectory (Barton, 1986). Consider the $t=0$ Gaussian state

$$\Psi(x,0) = \frac{1}{(\pi b^2)^{1/4}}\exp\left[-\frac{(x-x_0)^2}{2b^2}+\frac{i}{\bar{h}}p_0 x\right], \tag{S63}$$

centered at $x = x_0$ and where b is the distribution width. This profile minimizes the uncertainty principle (S52). In fact, the expectation values of position and momentum on this state are

$$\langle \hat{x} \rangle = \int_{-\infty}^{+\infty} dx\, x|\Psi(x,0)|^2 = x_0\,, \tag{S64a}$$

$$\langle \hat{x}^2 \rangle = \int_{-\infty}^{+\infty} dx\, x^2|\Psi(x,0)|^2 = x_0^2 + \frac{b^2}{2}\,, \tag{S64b}$$

$$\langle \hat{p} \rangle = -i\hbar \int_{-\infty}^{+\infty} dx\, \Psi^*(x,0)\partial_x\Psi(x,0) = p_0\,, \tag{S64c}$$

$$\begin{aligned}\langle \hat{p}^2 \rangle &= -\hbar^2 \int_{-\infty}^{+\infty} dx\, \Psi^*(x,0)\partial_x^2\Psi(x,0)\\ &= p_0^2 + \frac{\hbar^2}{2b^2}\,,\end{aligned} \tag{S64d}$$

so that an estimate of the size of quantum fluctuations in position is given by $\Delta x = \sqrt{\langle \hat{x}^2 \rangle - \langle \hat{x} \rangle^2} = b/\sqrt{2}$; then, $\Delta p = \sqrt{\langle \hat{p}^2 \rangle - \langle \hat{p} \rangle^2} = \hbar/(\sqrt{2}b)$ and

$$\Delta x \Delta p = \frac{\hbar}{2}\,. \tag{S65}$$

States saturating Heisenberg principle are called coherent. Calculating (S62) and taking the square, one finds the probability density

$$|\Psi(x,t)|^2 = \frac{1}{\sqrt{\pi B^2(t)}} \exp\left\{-\frac{[x - x(t)]^2}{B^2(t)}\right\}, \tag{S66}$$

where $x(t)$ is the classical trajectory (S48) and

$$B^2(t) = b^2 \cosh^2(\Omega t) + \frac{\hbar^2}{b^2\Omega^2} \sinh^2(\Omega t)\,. \tag{S67}$$

Thus, the wave packet maintains the Gaussian form and it saturates Heisenberg principle at all times. At short times, $B^2(t) = b^2 + (b^2\Omega^2 + \hbar^2 b^{-2})t^2 + O(t^4)$, while at late times $B^2(t) \simeq [b^2 + \hbar^2(b\Omega)^{-2}]e^{2\Omega t}/4$. Since B increases with t, the peak of probability follows the classical trajectory and it spreads exponentially as time goes by. Therefore, initially the system evolves almost classically (if b is small enough) and one can determine with good accuracy the association strength $v = \lambda - x$ of the subject, but at later trials there is a nonzero chance that the subject display a non-optimal conditioning level, even if the highest probability is at the asymptotic value $v = \lambda$. Quantum fluctuations increase from the initial value $b/\sqrt{2}$ to indefinitely large values $\Delta x = B(t)/\sqrt{2} \simeq be^{\Omega t}/2^{3/2}$, if $\hbar \ll b\Omega$. The probability density for the RW model is shown in **Figure S22**.

Prediction 3: There exists a quantum state such that response fluctuations are large and almost constant. A sharply peaked coherent state can be characterized by almost constant fluctuations. To see how, we make a few steps back and reflect on a basic property of the quantum model, doing a micro-analysis of the fluctuations. In order to understand the typical time scale involved in quantum fluctuations, one can calculate the sojourn time t_* in several but mutually agreeing ways which differ from one another by the level of rigorousness (Barton, 1986). Here we choose a semi-heuristic method based on an estimate of $\langle \hat{x}^2(t) \rangle$, the expectation value of the operator $\hat{x}^2$ in the so-called Heisenberg picture on a real-valued state $|\Psi_l\rangle$ that minimizes Heisenberg's uncertainty principle and maximizes t_* for a given length scale l.

In the Heisenberg picture, states are independent of t and operators evolve in time by the action of the quantum Hamiltonian. In particular, the position operator $\hat{x} = \hat{x}(0)$ becomes $\hat{x}(t) = e^{i\hat{H}t}\hat{x}e^{-i\hat{H}t}$, and a

similar expression holds for $\hat{p}(t)$. The criterion defining the sojourn time is established by the condition

$$\langle\Psi_l|\hat{x}^2(t)|\Psi_l\rangle \leqslant l^2\,, \qquad 0\leqslant t\leqslant t_*\,. \tag{S68}$$

The state $|\Psi_l\rangle$ is such that $\langle\hat{x}^2(0)\rangle = l^2$ [maximum value of $\langle\hat{x}^2(t)\rangle$ at $t=0$], $\langle\hat{x}^2(t)\rangle$ reaches a minimum at $t=t_*/2$ and grows again to l^2 at $t=t_*$. Regarding (S48) as the solution in the Heisenberg picture, we can replace it into (S68) and, if we also require $\Psi(x)=\langle x|\Psi\rangle$ to be real, get $\langle\hat{x}_0\hat{p}_0+\hat{x}_0\hat{p}_0\rangle=0$ and

$$\langle\Psi_l|\hat{x}_0^2\cosh^2(\Omega t)+\frac{\hat{p}_0^2}{\Omega^2}\sinh^2(\Omega t)|\Psi_l\rangle \leqslant l^2\,. \tag{S69}$$

A further assumption we make is that $|\Psi_l\rangle$ saturates Heisenberg principle (S53) (which is valid also in the Heisenberg picture), so that $\Delta x(t)\Delta p(t)=\bar{h}/2$ for all t. This is easily achieved by a wave packet analogous to (S63), but now with a width constant at all times. Concretely, a minimum-uncertainty state for the oscillator peaked at some $x=\bar{x}$ is the wave packet

$$\Psi_l(x)=\langle x|\Psi_l\rangle=\frac{1}{(\pi\bar{h}b_l^2)^{1/4}}\exp\left[-\frac{(x-\bar{x})^2}{2\bar{h}b_l^2}\right], \tag{S70}$$

where the constant b_l depends on l (here $\bar{h}b_l^2$ plays the role of b^2). In particular, for $\bar{x}=0$ calculations identical to those in (S64) yield $\langle\hat{x}(t)\rangle=0$, $\langle\hat{x}^2(t)\rangle=\bar{h}b_l^2/2$, $\langle\hat{p}(t)\rangle=0$, $\langle\hat{p}^2(t)\rangle=\bar{h}/(2b_l^2)$, so that Heisenberg principle is saturated for all t:

$$\langle\hat{x}^2(t)\rangle\langle\hat{p}^2(t)\rangle=\frac{\bar{h}^2}{4}\,, \tag{S71}$$

just as in the Schrödinger-picture relation (S65).

We can make a crude estimate of l by recalling that, classically, $x_0=\lambda$. Then, if the system is close to classicality at the beginning of the experiment, $l^2=\langle\hat{x}^2(0)\rangle=O(\lambda^2)$, where "$O$" means "of order of." Thus, the typical fluctuation has a size of order of the asymptote, $l=\delta v=O(\lambda)$.

Prediction 4: There is a universal constant $\bar{h}$ that can be determined by experiments. Saturating inequality (S69) at $t=t_*$ and using the identity $\cosh^2 z-\sinh^2 z=1$, we can invert (S69) and get

$$t_*=\frac{1}{2\Omega}\operatorname{arccosh} z\,, \qquad z=\frac{2l^2-\langle\hat{x}_0^2\rangle+\langle\hat{p}_0^2/\Omega^2\rangle}{\langle\hat{x}_0^2\rangle+\langle\hat{p}_0^2/\Omega^2\rangle}$$

where $\operatorname{arccosh} z=\ln(z+\sqrt{z^2-1})$ is the inverse hyperbolic cosine. Therefore, $z=(1+4l^2\Omega^2b_l^2/\bar{h}-\Omega^2b_l^4)/(1+\Omega^2b_l^4)$. Since $\operatorname{arccosh} z$ increases monotonically in z, t_* is maximized when z attains its maximum value $z=\sqrt{1+4l^4\Omega^2/\bar{h}^2}$ at $b_l^2=(\sqrt{1+4l^4\Omega^2/\bar{h}^2}-1)/(2l^2\Omega^2/\bar{h})$. Combining these expressions with the definition in (S9), the final result reads

$$t_*=\frac{1}{2\alpha\beta}\ln\left[\frac{2l^2\alpha\beta}{\bar{h}}+\sqrt{1+\frac{(2l^2\alpha\beta)^2}{\bar{h}^2}}\right]. \tag{S72}$$

The sojourn time increases monotonically with l: consistently with the first prediction, it takes longer to see larger quantum fluctuations. The greater the magnitude of the US, the greater t_*. This is somewhat intuitive: if the amount of US at each trial is high, the subject will tend to stay in the state of maximal conditioning longer. If we present the subject with 10 drops of saccharin solution, the state of maximal conditioning will decay *slower* than when presented with only one drop per trial.

However, for a fixed λ the sojourn time *decreases* with the saliences of the CS and of the US. This bizarre result may find a rationale if we admit that the total duration of the acquisition stage may affect the sojourn time at the asymptote of learning. Higher CS and US saliences lead to faster learning and the subjects reach

the asymptote early, thus increasing the chance of response fluctuations of the trials. Conversely, the longer the conditioning process the lower the chance for the subject to move away from optimal conditioning once reached.

The magnitude of this effect is determined by the constant $\bar{h}$, which we assumed to be independent of the stimuli. Let us consider trial-by-trial individual data, which are those with better time resolution. The constant $\bar{h}$ (the analogue of Planck constant $\hbar$ in quantum mechanics) is an external input that can be determined experimentally once the other parameters are known. While data allow us to find the best-fit values of $\alpha\beta$ and λ, we only have an upper limit on t_* coming from the fact that responses vary considerably already from one trial to the next:

$$t_* \lesssim \Delta t_{\rm trial} = 1\,, \tag{S73}$$

which translates into a lower bound on $\bar{h}$. To find this bound, we must approximate Eq. (S72). The values of $\alpha\beta$ can be read from **Table S1** for session-by-session data and from **Table S2** for trial-by-trial data. In the first case, $\alpha\beta \sim 10^{-2} - 10^{-1}$, while in the second it is even smaller, $\alpha\beta \sim 10^{-3} - 10^{-2}$. Therefore, the approximation $\alpha\beta \ll 1$ holds regardless of the magnitude of the ratio $\bar{h}/l^2$. If this ratio is of order unity, an expansion of Eq. (S72) in $\alpha\beta \ll 1$ yields $t_* \simeq 2l^2\alpha\beta/\bar{h}$, hence $\bar{h} \simeq 2l^2\alpha\beta/t_* \gtrsim 2l^2\alpha\beta$. If instead $\bar{h} \ll l^2$, an expansion in $l^2\alpha\beta/\bar{h} \gg 1$ gives $t_* \simeq (2\alpha\beta)^{-1}\ln(2l^2\alpha\beta/\bar{h})$, hence $\bar{h} \simeq 2l^2\alpha\beta\exp(-2\alpha\beta t_*) \gtrsim 2l^2\alpha\beta\exp(-2\alpha\beta) \simeq 2l^2\alpha\beta$. In both cases, we find the same lower limit (S77), which is also consistent with the following argument. We can get a simple estimate of $\bar{h}$ by assuming that the Gaussian wave packet is an appropriate description of the association strength (v) of the subject. As said above, this state saturates the uncertainty principle, Eq. (S65). On the other hand, we have not seen an appreciable spread of the distribution of v values during the experiment, which means that the wave packet is sharply peaked at the classical trajectory. Therefore, we can take the classical evolution equation $\dot{x} = -\alpha\beta x$ (Eq. (2) in the main paper) and the classical momentum (S45) ($p = \dot{x} = -\alpha\beta x$) to get a rough estimate of the momentum fluctuations $\Delta p \sim \alpha\beta\Delta x$ (here absolute values are understood), so that Eq. (S65) implies (S77): $\bar{h} \sim 2\alpha\beta(\Delta x)^2 \sim 2\alpha\beta l^2 \sim 2\alpha\beta\sigma^2$.

Prediction 5: Response variability is described by white noise. The spectrum of the quantum fluctuations described by the state (S70) is Gaussian. In fact, a Gaussian distribution is such that all even-order correlations can be expressed in terms of the two-point correlation function (Adler, 1981; Adler and Taylor, 2007). In our case, odd higher-order momenta vanish identically $\langle \hat{x}^{2n+1}\rangle = 0$, while even momenta are

$$\langle \hat{x}^{2n}\rangle = (2n-1)!!\left(\frac{\bar{h}b_l^2}{2}\right)^n = (2n-1)!!\langle \hat{x}^2\rangle^n, \tag{S74}$$

where "!!" is the double factorial: $N!! = N(N-2)(N-4)\cdots$. Thus, $n = 1$ is the second-order correlation (S64b) (with $x_0 = 0$ and $b^2 = \bar{h}b_l^2$), $\langle \hat{x}^4\rangle = 3\langle \hat{x}^2\rangle^2$, and so on. A Gaussian distribution of random fluctuations is precisely white noise.

6.3 Summary of the theory

In the classical RW model, at time t we can determine with absolute accuracy the strength $v(t)$ and, from $\dot{v} = \alpha\beta(\lambda - v)$ (Eq. (2) in the main paper), the velocity of learning or learning rate $\dot{v}(t) = \alpha\beta[\lambda - v(t)]$. After quantizing this model with the procedure described in section 6.2, as a result we can no longer determine $v(t)$ and $\dot{v}(t)$ with arbitrary accuracy. Curiously, the uncertainty Δv on the association strength v is limited by the uncertainty $\Delta\dot{v}$ on the learning rate $\dot{v}$, in such a way that, in general, if we measure v with very good accuracy there will be a large uncertainty on a $\dot{v}$ measurement, and vice versa. In other words, if we force Δv to get as close to zero as possible, then $\Delta\dot{v}$ will increase, and vice versa. This relative minimum uncertainty, present even in an error-free ideal experiment, is governed by the relation

$$\Delta v\Delta\dot{v} \geqslant \frac{\bar{h}}{2}\,, \tag{S75}$$

where $\bar{h}$ is a strictly positive constant, a fundamental parameter of the theory. In the classical model, $\bar{h} = 0$ and we can measure v and $\dot{v}$ with infinite accuracy (in an error-free experiment) simultaneously.

A priori, the subject has infinitely many ways to reach optimal learning $v = \lambda$ starting from complete ignorance $v = 0$ at $t = 0$. While in the classical model there exists only one single learning curve $v(t)$

connecting the points $v(0) = 0$ and $v(\text{large}\, t) \simeq \lambda$, in the quantum model all the possible trajectories are realized simultaneously and in such a way that they interfere with one another. The net effect is the uncertainly relation (S75). The reader may consult section 6.2 for more details about this perspective, which goes under the name of path-integral quantization.

The quantum model gives rise to several prediction.

- *Prediction 1.* The asymptote of learning is not a durable achievement. There exists an intrinsic source of variability in the subject response, not due to statistical errors or unpredictability of individual behavioral quirks. Only three situations as possible: (i) response variability is too slow to be detected, (ii) it only happens between training sessions, or (iii) it happens on a trial-by-trial basis.
- *Prediction 2.* There exists a special quantum state following a Gaussian distribution such that response fluctuations do not decrease in time. Initially, the system evolves almost classically and one can determine with good accuracy the association strength of the subject, but at later trials there is a nonzero chance that the subject display a nonoptimal conditioning level, even if the highest probability is at the asymptotic value $v = \lambda$. Quantum fluctuations increase to indefinitely large values.
- *Prediction 3.* There exists a quantum state such that response fluctuations are large and almost constant throughout the whole experiment. This state minimizes the uncertainly principle (S75), so that $\Delta v \Delta \dot{v} = \bar{h}/2$ at all times. The amplitude δv of quantum fluctuations is of order of the uncertainty Δv, which in turn is of order of the classical asymptote of learning:
$$\Delta v \sim \delta v = O(\lambda)\,. \tag{S76}$$
- *Prediction 4.* The universal constant $\bar{h}$ can be determined by experiments and reads
$$\bar{h} \simeq 2\alpha\beta(\Delta v)^2\,. \tag{S77}$$
- *Prediction 5.* Response variability is described by white noise.

Again, we refer to Appendix 6.2 for a top-down derivation of these claims. Here we comment on a central aspect relevant to their empirical check. The first prediction is a consequence of the inequality (S75). At any given time (i.e., trial), the uncertainty Δv on the association strength can be interpreted as the fact that the association between the CS and the US continuously varies in strength and is subject to what we may call *quantum fluctuations*. After reaching the asymptote at $v = \lambda$, the subject will not keep the maximal response λ because, due to these quantum fluctuations, the association strength will be shifted by some amount $\delta v > 0$. Therefore, the actual response at any given time fluctuates as $\lambda \pm \delta v$. After some characteristic *sojourn time* t_* since reaching the point $v = \lambda$, the subject shows signs of "unlearning" (if the fluctuation is negative and $\lambda \to \lambda - \delta v$) or even of "overlearning" (if the fluctuation is positive and $\lambda \to \lambda + \delta v$)!

Therefore, at times $t > t_*$, the subject can display either of two behaviors: (A) it loses the incentive even if the US is presented at each subsequent trial and the probability to show a conditioned response upon presentation of the CS starts to decrease; or, (B) its response increases beyond the learning asymptote. Neither situation (A) nor (B) lasts forever because once the particle rolls down back or across the hill-top (under- or over-response by the subject), the presentation of new trials pushes the association strength back to the asymptote. Once reached the asymptote again at a time $t_1 > t_*$, the subject will stay there for another period of duration $\sim t_*$, after which another fluctuation will happen at a time $\sim t_* + t_1$ since the beginning of the experiment. And so on.

What do we expect to observe then? In an ideal experiment of quantum physics, one prepares the particle at the unstable maximum at time $t = 0$ and asks what the probability is to find the particle at the same state after some time t. However, pointwise measurements and continuous observation of unstable systems (Fonda et al., 1978) can give rise to very different outcomes. Persistence at the maximum can be extended if the system is measured too often or continuously (i.e., observed) and not left to evolve naturally a sufficiently long time (Sudbery, 1984). If one observes the system at a time $t < t_*$, most likely one will not detect any change with respect to the initial state, while at times $t > t_*$ decay from the unstable state will be observed more likely. In the context of comparative psychology, this distinction is less clear. The natural time evolution of the system corresponds to unobservable cognitive processes taking place in the subject after training, away from the experimenter and the conditioning box, while measuring the response of the

subject in an experiment yields an empirical estimate of the otherwise unobservable associative strength. However, separating the natural evolution from measurement is a tricky business because "measuring" also means making the system evolve by training. One might change interpretation and insist that "natural evolution" is equivalent to training and a "measurement" corresponds to testing the result of training at the last trial, but this distinction would be quite artificial because the measurement procedure does not depend on whether the US is presented or not. Therefore, each time the subject is trained the association strength evolves *and* is measured at the same time. As a consequence, we can identify three situations, depending on whether t_* is greater or smaller than the inter-trial and inter-session intervals.

- (i) $t_* > \Delta t_{\rm session} > \Delta t_{\rm trial}$. If t_* is greater than the duration of a trial $\Delta t_{\rm trial}$, the phenomenon of sojourn time is altered, and in fact reset, each time a break in conditioning is taken. In this case, once the subject reaches the asymptote of learning we should observe a constant response at $v = \lambda$, at all trials. If t_* is also greater than the inter-session interval, then the response is stable both on a trial-by-trial and on a session-by-session time scale. The learning curve would be identical to the classical one in both time scales.
- (ii) $\Delta t_{\rm trial} < t_* < \Delta t_{\rm session}$. If t_* were greater than the trial duration $\Delta t_{\rm trial}$ but smaller than the inter-session interval $\Delta t_{\rm session}$, then we should observe a change in response between sessions, rapidly restored to maximal learning after the first trials of the session. The trial-by-trial learning curve would then display a long plateau marked by local "pulses" or fluctuations lasting only the first few trials of each session.
- (iii) $t_* < \Delta t_{\rm trial}$. If t_* is smaller than the duration of a trial, then we should observed decay, to the left (under-response) or to the right (over-response), from the learning asymptote. The overall picture after the acquisition phase is neither that of a constant, stable maximal response (as in the classical RW model or when $t > \Delta t_{\rm session}$) nor a sudden decrease or increase in the response some time after reaching the asymptote (as it would happen in a physics experiments), nor a sudden decrease or increase of response at the beginning of each session (as when $\Delta t_{\rm trial} < t < \Delta t_{\rm session}$). Rather, there would be a never-ending series of trial-by-trial oscillations above and below the asymptote.

6.4 Empirical evidence of predictions

1. Our observations and those of Harris et al. (2015) clearly fall into case (iii). Response variations occur on a trial-by-trial time scale $t_* < \Delta t_{\rm trial}$.
2. If the system were described by a Gaussian state, response fluctuations should increase in time. This prediction is not met. If one starts from a nearly classical state with a spread width b close to zero, late-time fluctuations may become compatible with data, but then early variations of the response are too small relative to those actually observed. Roughly speaking, the problem is that, regardless the absolute size of the fluctuations, we did not observe any relative change in them during the experiment. There are two alternative meanings we can attach to this outcome. Either the Gaussian state is a valid description but b is so small (i.e., the initial state is so sharply peaked at the classical solution) that we cannot see an appreciable spread of the distribution of the association strength v in the total time scale of the experiment; or we must exclude the Gaussian state (S63) as a viable initial condition. The second possibility does not rule out the theory as a whole because one can choose other states, usually constructed from the non-normalizable energy eigenstates of the inverted oscillator (see Barton, 1986). In general, however, these will not minimize the uncertainty principle, and they will suffer from the same problem of fast spreading in time. Due to the quantum uncertainty in the rate of change of the association strength, the association strength can acquire different velocities and so get further and further displaced from the classical value.
3. The prediction (S76) is compatible with what we found in our experiment and in Experiment 2 of Harris et al. (2015). Identifying the amplitude of fluctuations with the estimated standard deviation of the fits with the RW model,

$$\delta v \stackrel{?}{=} \sigma \,, \tag{S78}$$

in our long experiment we get $\sigma/\lambda = 0.1 - 0.6$: the uncertainty of the association strength is between 10% and 60% the value of the asymptote, depending on the subject, for an average of about one third of the asymptote. This is true not only for the session-by-session data (**Table 1** in the main paper), but also for the trial-by-trial ones (**Table S2**). Using trial-by-trial data, the average of the variance σ^2 for

each group and for all experimental subjects together is

$$\text{Group 1:} \qquad l^2 \simeq \sigma^2_{\text{avg}} = 129.2 \pm 40.5\,, \tag{S79a}$$

$$\text{Group 2:} \qquad l^2 \simeq \sigma^2_{\text{avg}} = 154.0 \pm 63.9\,, \tag{S79b}$$

$$\text{Group 1+2:} \qquad l^2 \simeq \sigma^2_{\text{avg}} = 142.5 \pm 55.6\,. \tag{S79c}$$

The value obtained in each group separately is the same within the experimental error. We can improve this result by recalling that the quantum model we are testing is based on the inverted harmonic oscillator associated with the RW classical model. However, we saw that at least subjects 1-7, 2-3, 2-4, and 2-8 follow an oscillatory learning curve. Therefore, strictly speaking the quantum RW model does not apply to these animals. On the other hand, developing a quantum theory over the DOM (Eq. (8) in the main paper) can be more difficult than for the RW model (S2) because one cannot recast the model as a simple inverted harmonic oscillator and one has to deal with the dissipative forces of a damped model. As a simple way to obviate this problem, we discard the data of subjects 1-7, 2-3, 2-4, and 2-8 from the estimates of $\hbar$. The result is a decrease in the error uncertainty:

Group 1 (no 1-7):

$$l^2 \simeq \sigma^2_{\text{avg}} = 139.8 \pm 33.6\,, \tag{S80a}$$

Group 2 (no 2-3, 2-4, 2-8):

$$l^2 \simeq \sigma^2_{\text{avg}} = 123.2 \pm 59.7\,, \tag{S80b}$$

Group 1+2 (no 1-7, 2-3, 2-4, 2-8):

$$l^2 \simeq \sigma^2_{\text{avg}} = 132.3 \pm 48.0\,. \tag{S80c}$$

Thus, the typical response oscillation is similar for all subjects and is large despite the quantum state being very close to classicality.

For Harris et al. (2015) Experiment 2, we found the averaged values of σ and λ given in **Table S5**. In this table, we split the data set into subjects following RW and those with oscillations. Strictly speaking, the quantum RW model should be tested only for those subjects that have RW as the best fit, but for completeness we include also the other subjects separately. Considering only RW subjects, we find $\sigma/\lambda = 0.2 - 0.4$, compatible with the prediction of the model and with the range found in the other experiment.

4. Applying the estimate (S78) to Eq. (S77) yields $\hbar \simeq 2\alpha\beta\sigma^2$. This expression is crude and can only give generic qualitative indications about the viability of the model. Plugging the values of $\alpha\beta$ and σ^2 for each subject individually, we get a rather heterogeneous set of values, reported in **Table S6**.

   One of the data is especially off the average, namely, subject 2-5. This is due to its extremely high value of $\alpha\beta \approx 0.77$. Removing this datum, the average of Group 2 is comparable with that of Group 1. The grand average is 1.7 ± 1.3. The uncertainty is of order of the central value and, although the latter is different from zero within one standard deviation, it does not constitute a compelling evidence of the validity of the model.

   For Harris et al. (2015) Experiment 2, taking the best-fit values of the parameters $\alpha\beta$ and λ only for subjects following RW (we will report these values in Calcagni et al. (2019)), we found the estimates of $\hbar$ listed in **Table S7**.

   Within each group, the estimated average of $\hbar$ is of the same order as its error, which means that the range of values is quite dispersed, at least more than in the long experiment where the average $\hbar$ is nonzero significantly. Therefore, the conclusion is that also Experiment 2 of Harris et al. (2015) is unable to verify this prediction of the quantum model. The latter would be ruled out if the group values would have been significantly different from one another.

5. Recalling that there is no trace of color in the data and that the descriptive stochastic model with white noise is the best fit, we immediately conclude that the quantum theory, which also predicts a white-noise spectrum of fluctuations, adequately describes the signal of all subjects.

6.5 Discussion

Holden et al. (2011) and Van Orden et al. (2010) suggested the hypothesis that response variability could be analogous to that produced by quantum fluctuations in quantum mechanics. This parallelism was partially motivated by the fact that human behavior can be affected by its measurement, just like a quantum system is modified by the act of measuring with an apparatus. Here we took the analogy *ad litteram* and, after having defined the quantum system rigorously, checked whether it is experimentally viable. We found that response variability *could* be interpreted as quantum fluctuations of the size

$$\delta v \sim \sqrt{\frac{\bar{h}}{2\alpha\beta}}\,. \tag{S81}$$

Any other model predicting an $\alpha\beta$-dependent δv with a different trend in (and magnitude of) the amplitude of fluctuations would hardly find a subject-independent constant $\bar{h}$ to a significant confidence level. Combining the estimate on $\bar{h}$ with the prediction on white noise, we can conclude that data *are* compatible with the quantum theory. However, we cannot go beyond a moderate optimism regarding its validity, since the error associated with the estimates (S79) is rather large, about 30 to 40% of the value. The error in the corrected estimates (S80) is slightly smaller. This uncertainty is due to the estimation of the amplitude of fluctuations by the rough guess (S78). Moreover, statistical white noise can obviously accommodate data with a flat stochastic signal. Another potential source of white noise is the experimental design itself. The delivery of the US with a uniform distribution during the CS already introduces a random component that should reflect in the subjects' response. Therefore, one would expect white noise even in the total absence of any "cognitive" or "quantum" mechanism. This random component is already taken into account in the statistical error, which, as we just noted, dominates the relation (S77) and the estimates (S79) and (S80).

So far, we ignored the fact that $\bar{h}$ is a dimensionful constant. All values of $\bar{h}$ should be quoted in units of (association strength)^2 per time unit or, more specifically, "(licks)^2/trial" in the case of the long experiment, and "(head entries)^2/trial" in the case of Experiment 2 of Harris et al. (2015). Clearly, these units become cumbersome when trying to compare values obtained in different experiments with different dependent variables and trial duration. At any rate, the priority should be to verify the constancy of $\bar{h}$ with independent data, leaving the cross-experimental determination of the actual value of $\bar{h}$ as a next-step problem. In particular, one should investigate whether response fluctuations remain large, constant, and with a white-noise spectrum also when the US/reinforcer is presented on a more regular strictly deterministic schedule.

To summarize, we have analyzed the quantum theory using the RW model as the classical layer and found that it could describe response fluctuations, although we could not find conclusive evidence. Quantizing the DOM will probably clarify this open question. The psychological interpretation of the quantum theory remains open. The idea that quantum mechanics can play a role in brain processes and in the emergence of consciousness is almost as old as the discipline (see Stanford Encyclopedia of Philosophy, 2015, for an account) and fostered debate among psychologists, physicists and philosophers, sometimes leading to original collaborations between authorities in their respective fields (Beck and Eccles, 1992; Bohm, 1990, 2002; Jung and Pauli, 1952; Penrose, 1989; Ricciardi and Umezawa, 1967; Stapp, 2009; Wigner, 1967). Recently, there has been interest in applying quantum mechanics to human cognition and decision making (Bruza et al., 2009a; 2015; Busemeyer and Bruza, 2012; Busemeyer et al., 2006; 2009; 2011; 2014; Pothos and Busemeyer, 2011; Pothos et al., 2017; Trueblood and Busemeyer, 2012; Yearsley and Busemeyer, 2015), reinforcement learning (Fakhari el al., 2013), as well as in the general field of artificial intelligence (Bruza et al., 2009b; Busemeyer et al., 2012). The role of quantum physics at the time scale of brain processes ($\sim 10-100$ ms) is still controversial (Hagan et al., 2002; Tegmark, 2000) and it is not clear whether and how it affects cognition and behavior. Our main concern here is not the origin of quantum cognitive or behavioral phenomena, but the collection of empirical evidence (or the lack of it) in their favor in the context of animal learning, novel with respect to the above literature. We may come back to the psychological interpretation of the quantum RW model only after better evidence is gathered. Our results regarding this model are insufficient to validate it but, at the same time, we hope they are encouraging enough to warrant further research.

REFERENCES

Adler, R.J. (1981). *The Geometry of Random Fields*. London, UK: Wiley.

Adler, R.J., & Taylor, J. (2007). *Random Fields and Geometry*. New York, NY: Springer.

Barton, G. (1986). Quantum mechanics of the inverted oscillator potential. *Ann. Phys. (N.Y.) 166*, 322.

Beck, F., & Eccles, J. (1992). Quantum aspects of brain activity and the role of consciousness. *Proc. Nat. Acad. Sci. USA 89*, 11357.

Bernal, S.Y., Dostova, I., Kest, A., Abayev, Y., Kandova, E., Touzani, K., Sclafani, A., & Bodnar, R.A. (2008). Role of dopamine D1 and D2 receptors in the nucleus accumbens shell on the acquisition and expression of fructose-conditioned flavor–flavor preferences in rats. *Behav. Brain Res. 190*, 59.

Bian, X., Tu, P., Chi, L., Gao, B., Ru, H., & Lu, K. (2017). Saccharin induced liver inflammation in mice by altering the gut microbiota and its metabolic functions. *Food Chem. Toxicol. 107*, 530.

Bohm, D. (1990). A new theory of the relationship of mind and matter. *Philos. Psychol. 3*, 271.

Bohm, D. (2002). *Wholeness and the Implicate Order*. Hoboken, NJ: Routledge.

Bruza, P., Busemeyer, J.R., & Gabora, L. (2009). Introduction to the special issue on quantum cognition. *J. Math. Psychol. 53*, 303.

Bruza, P., Sofge, D., Lawless, W., van Rijsbergen, C.J., & Klusch, M. (Eds.) (2009). *Quantum Interaction. Third International Symposium, QI 2009*. Heidelberg, Germany: Springer-Verlag

Bruza, P.D., Wang, Z., & Busemeyer, J.R. (2015), Quantum cognition: a new theoretical approach to psychology. *Trends Cog. Sci. 19*, 383.

Busemeyer, J.R., & Bruza, P.D. (2012). *Quantum Models of Cognition and Decision*. Cambridge, UK: Cambridge University Press.

Busemeyer, J.R., Pothos, E.M., Franco, R., & Trueblood, J.S. (2011). A quantum theoretical explanation for probability judgment errors. *Psychol. Rev. 118*, 193.

Busemeyer, J.R., Dubois, F., Lambert-Mogiliansky, A., & Melucci, M. (2012). *Quantum Interaction. Sixth International Symposium, QI 2012*. Heidelberg, Germany: Springer-Verlag

Busemeyer, J.R., Wang, Z., Khrennikov, A., & Basieva, I. (2014). Applying quantum principles to psychology. *Phys. Scr. T163*, 014007.

Busemeyer, J.R., Wang, Z., & Lambert-Mogiliansky, A. (2009). Empirical comparison of Markov and quantum models of decision making. *J. Math. Psychol. 53*, 423.

Busemeyer, J.R., Wang, Z., & Townsend, J.T. (2006). Quantum dynamics of human decision-making. *J. Math. Psychol. 50*, 220.

Calcagni, G. (2018). The geometry of learning. *J. Math. Psychol. 84*, 74 [`arXiv:1605.00591`].

Calcagni, G., Harris, J., & Pellón, R. (2019). Beyond Rescorla–Wagner: The ups and downs of learning. Work in progress.

Fakhari, P., Rajagopal, K., Balakrishnan, S.N., & Busemeyer, J.R. (2013). Quantum inspired reinforcement learning in changing environment. *New Math. Nat. Comp. 9*, 273.

Fonda, L., Ghirardi, G.C., & Rimini, A. (1978). Decay theory of unstable quantum systems. *Rep. Prog. Phys. 41*, 587.

Fujita, Y., Wideman, R.D., Speck, M., Asadi, A., King, D.S., Webber, T.D., Haneda, M., & Kieffer, T.J. (2009). Incretin release from gut is acutely enhanced by sugar but not by sweeteners in vivo. *Am. J. Physiol. Endocrinol. Metab. 296*, E473.

Gallistel, C.R. (2012). On the evils of group averaging: Commentary on Nevin's "Resistance to extinction and behavioral momentum.". *Behav. Proc. 90*, 98.

Gallistel, C.R., Fairhurst, S., & Balsam, P. (2004). The learning curve: implications of a quantitative analysis. *Proc. Nat. Acad. Sci. USA 101*, 13124.

Hagan, S., Hameroff, S.R., & Tuszyński, J.A. (2002). Quantum computation in brain microtubules: decoherence and biological feasibility. *Phys. Rev. E 65*, 061901 [`arXiv:quant-ph/0005025`].

Harris, J.A., Patterson, A.E., & Gharaei, S. (2015). Pavlovian conditioning and cumulative reinforcement rate. *J. Exp. Psychol. Anim. Learn. Cogn. 41*, 137.

Haubold, H.J., Mathai, A.M., & Saxena, R.K. (2001). Mittag-Leffler functions and their applications. *J. Appl. Math. 2011*, 298628 [`arXiv:0909.0230`].

Holden, J.G., Choi, I., Amazeen, P.G., & Van Orden, G. (2011). Fractal $1/f$ dynamics suggest entanglement of measurement and human performance. *J. Exp. Psychol. Hum. Percept. Perform. 37*, 935.

Hull, C.L. (1943). *Principles of Behavior*. New York, NY: Apple-Century-Crofts.

Jung, C.G., & Pauli, W. (1955). *Naturerklärung und Psyche*. Zürich, Switzerland: Rascher. Translated by P. Silz in *The Interpretation of Nature and the Psyche*. New York, NY: Pantheon.

Kemble, E.C. (1935). A contribution to the theory of the B. W. K. method. *Phys. Rev. 48*, 549.
Kilbas, A.A., Srivastava, H.M., & Trujillo, J.J. (2006) *Theory and Applications of Fractional Differential Equations*. Amsterdam, The Netherlands: Elsevier.
Mackintosh, N.J. (1975). A theory of attention: Variations in the associability of stimuli with reinforcement. *Psychol. Rev. 82*, 276.
Penrose, R. (1989). *The Emperor's New Mind*. Oxford, UK: Oxford University Press
Pothos, E.M., & Busemeyer, J.R. (2011). Formalizing heuristics in decision-making: a quantum probability perspective. *Front. Psychol. 2*, 289.
Pothos, E.M., Busemeyer, J.R., Shiffrin, R.M., & Yearsley, J.M. (2017). The rational status of quantum cognition. *J. Exp. Psychol. Gen. 146*, 968
Rescorla, R.A., & Wagner, A.R. (1972). A theory of Pavlovian conditioning: variations in the effectiveness of reinforcement and nonreinforcement. In A.H. Black & W.F. Prokasy (Eds.), *Classical Conditioning II* (pp. 64–99). New York, NY: Appleton-Century-Crofts.
Ricciardi, L.M., & Umezawa, H. (1967). Brain and physics of many-body problems. *Kibernetik 4*, 44.
Sclafani, A., & Ackroff, K. (1994). Glucose- and fructose-conditioned flavor preferences in rats: taste versus postingestive conditioning. *Physiol. Behav. 56*, 399.
Shimbori, T. (2000). Operator methods of the parabolic potential barrier. *Phys. Lett. A 273*, 37 [arXiv:quant-ph/9912073].
Shimbori, T., & Kobayashi, T. (2000). Complex eigenvalues of the parabolic potential barrier and Gel'fand triplet. *Nuovo Cim. B* **115**, 325 (2000) [arXiv:math-ph/9910009].
Sidman, M. (1952). A note on functional relations obtained from group data. *Psychol. Bull. 49*, 263.
Stanford Encyclopedia of Philosophy (2015). https://plato.stanford.edu/entries/qt-consciousness.
Stapp, H.P. (2009). *Mind, Matter, and Quantum Mechanics*. Berlin, Germany: Springer
Swithers, S.E., & Hall, W.G. (1994). Does oral experience terminate ingestion?. *Appetite 23*, 113.
Tegmark, M. (2000). Importance of quantum decoherence in brain processes. *Phys. Rev. E 61*, 4194 [arXiv:quant-ph/9907009].
Trueblood, J.S., & Busemeyer, J.R. (2012). A quantum probability model of causal reasoning. *Front. Psychol. 3*, 138.
Sudbery, A. (1984). The observation of decay. *Ann. Phys. (N.Y.) 157*, 512.
Van Orden, G.C., Kello, C.T., & Holden, J.G. (2010). Situated behavior and the place of measurement in psychological theory. *Ecol. Psychol. 22*, 24.
Wagner, A.R., & Rescorla, R.A. (1972). Inhibition in Pavlovian conditioning: applications of a theory. In M.S. Halliday & R.A. Boakes (Eds.), *Inhibition and Learning* (pp. 301–336). London, UK: Academic Press.
Wigner, E.P. (1967). Remarks on the mind-body question. In E.P. Wigner (Ed.), *Symmetries and Reflections* (pp. 171–184). Bloomington, IN: Indiana University Press.
Yearsley, J.M., & Busemeyer, J.R. (2015). Quantum cognition and decision theories: a tutorial. *J. Math. Psychol. 74*, 99.
Yuce, C., Kilic, A., & Coruh, A. (2006). Inverted oscillator. *Phys. Scr. 74*, 114 [arXiv:quant-ph/0703234].

FIGURES AND TABLES

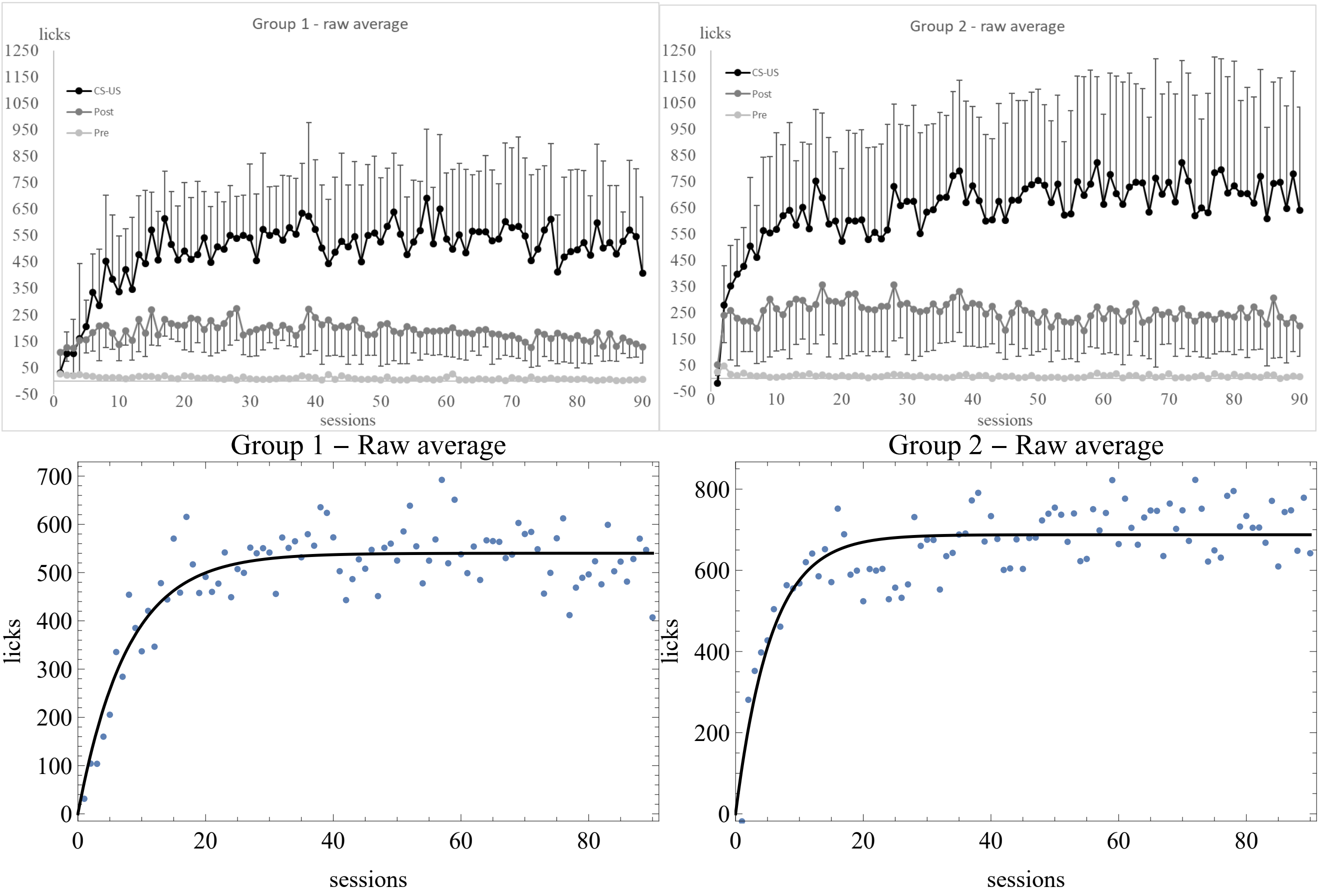

Figure S1. Average of raw (non-normalized) data, for Group 1 (top left) and 2 (top right), together with the best fit with the RW model for Group 1 (bottom left) and 2 (bottom right). Sessions are on the *horizontal axis*, number of licks on the *vertical axis*. Light gray, dark gray, and black data points (connected by lines of the same colors) are the licks in the 10 s respectively before, during, and after the CS. The number of US has been subtracted from the during-CS licks of each individual before taking the average, and the upper and lower error bars at the 68% confidence level of CS and post-CS data are shown.

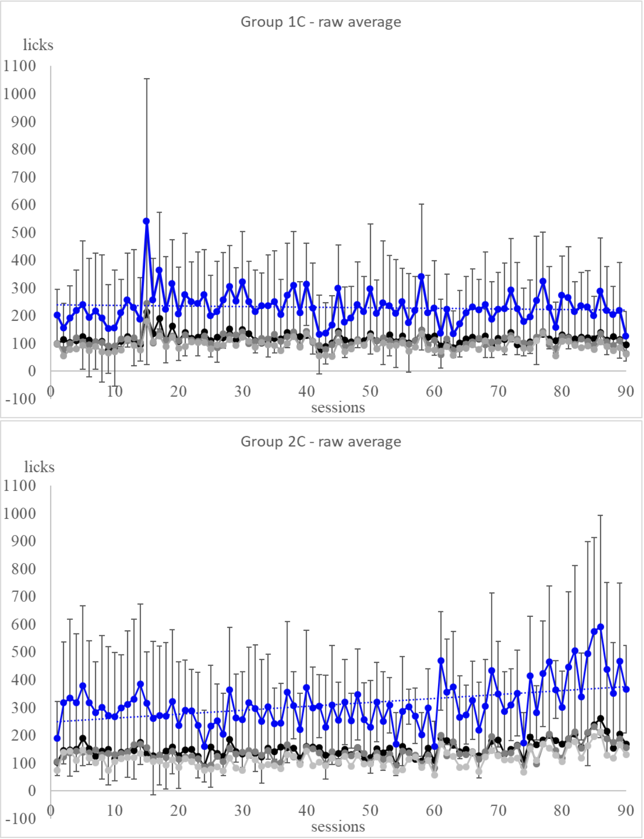

Figure S2. Average of raw (non-normalized) data, for Group 1C (top) and 2C (bottom). Sessions are on the *horizontal axis*, number of licks on the *vertical axis*. Light-gray, dark-gray, and black data are, respectively, pre-CS, CS, and post-CS licks, *without* US subtraction. As an added elaboration of the data, we also show the sum of pre-CS, CS, and post-CS licks (blue trendline; darkest gray in B/W rendering), where the number of US has been subtracted from the total licks of each individual before taking the average, and the upper and lower error bars at the 68% confidence level of CS and post-CS data are shown. The dashed line is the regression line of the “summed” data.

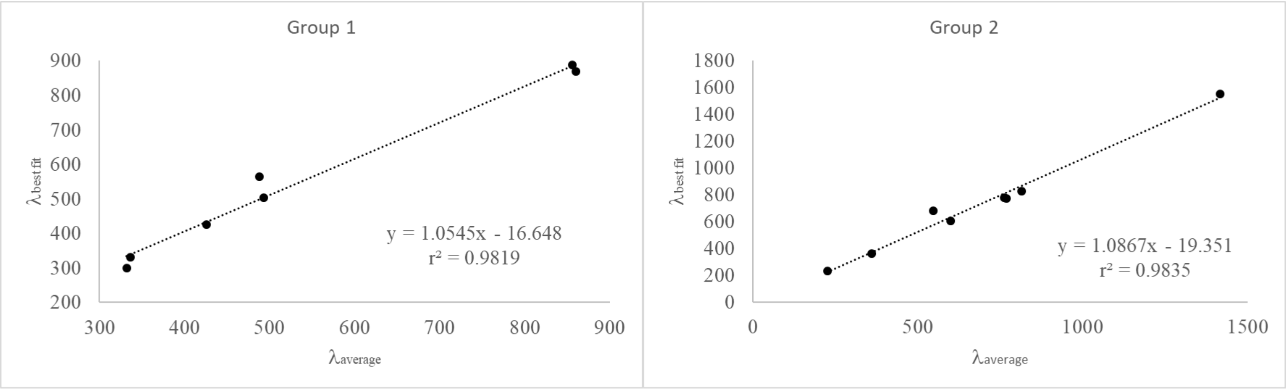

Figure S3. Linear regression between λ_{average} determined by the averaging procedure and $\lambda_{\text{best fit}}$ determined by the best-fit procedure, for Group 1 (left) and 2 (right). The linear equation with r^2 is shown.

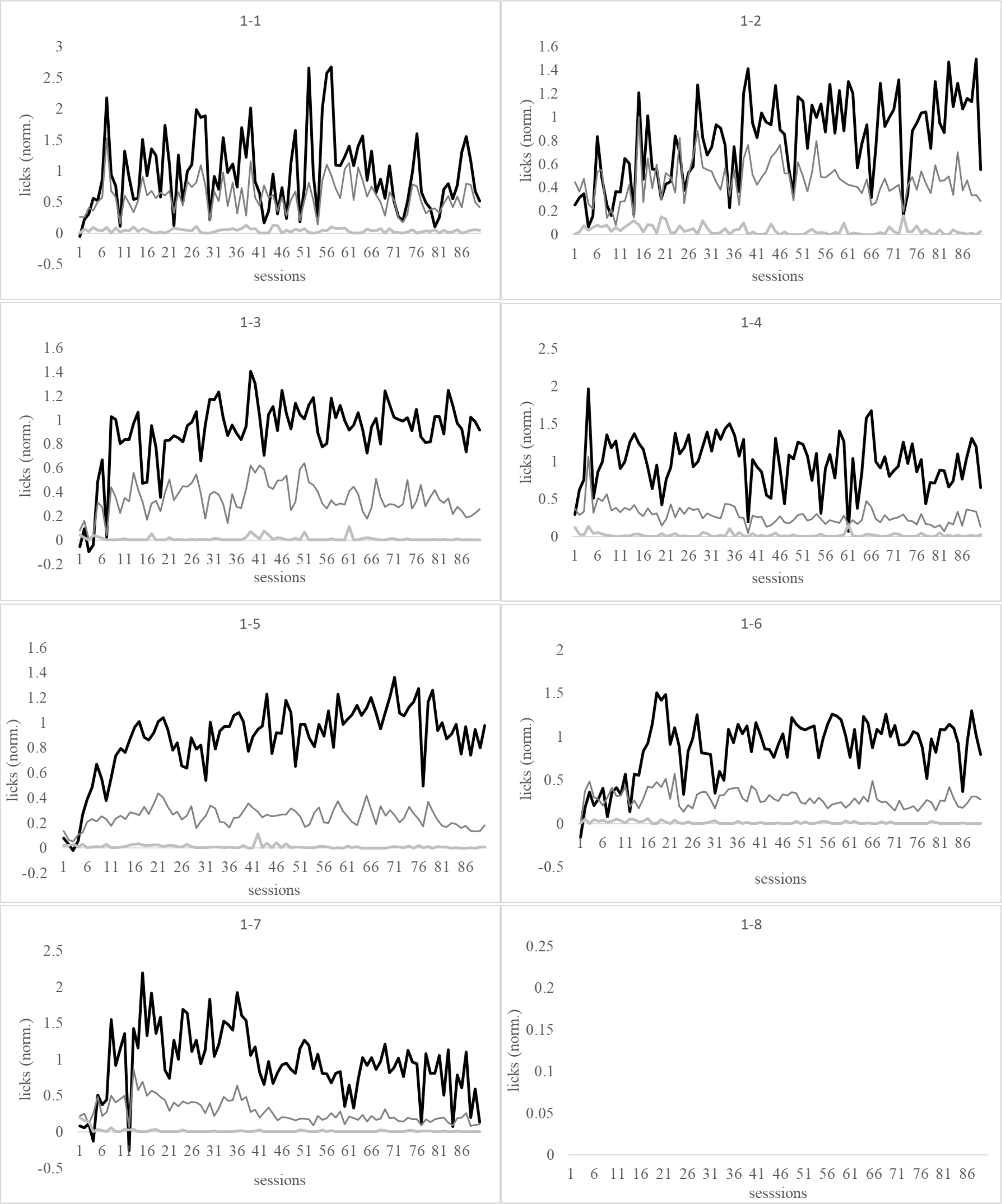

Figure S4. Individual normalized data of Group 1: licks before (light gray trend), during (black trend) and after (dark gray trend) the CS, divided by the best-fit λ. The number of US has been subtracted to the licks during the CS. Sessions are on the *horizontal axis*, the normalized response is on the *vertical axis*. Subject 1-8 was removed from the analysis.

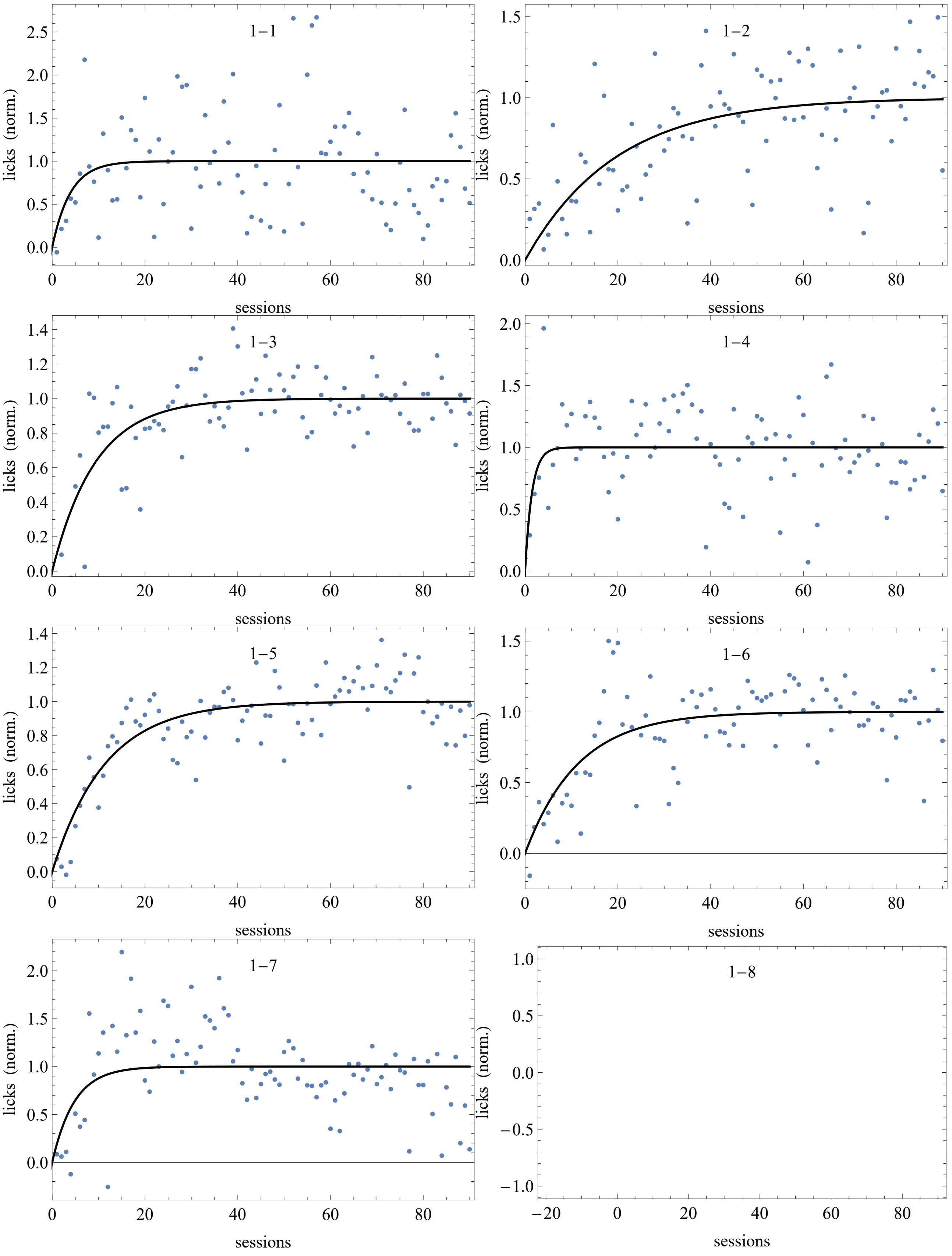

Figure S5. Individual normalized data (CS-licks minus US) of Group 1 with the best RW-model fit. Sessions are on the *horizontal axis*, the normalized response is on the *vertical axis*. Subject 1-8 was removed from the analysis.

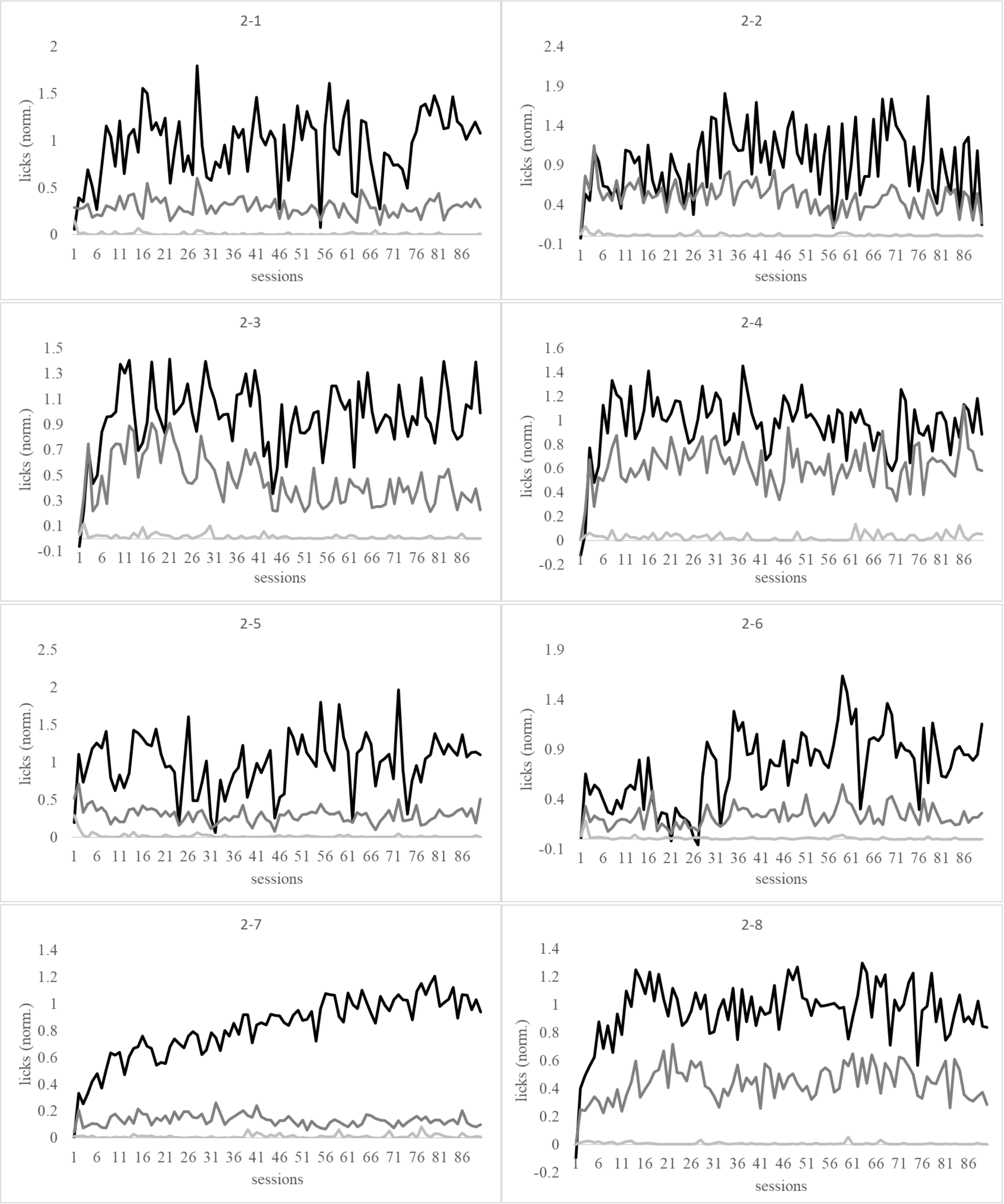

Figure S6. Individual normalized data of Group 2: licks before (light gray trend), during (black trend) and after (dark gray trend) the CS, divided by the best-fit λ. The number of US has been subtracted to the licks during the CS. Sessions are on the *horizontal axis*, the normalized response is on the *vertical axis*.

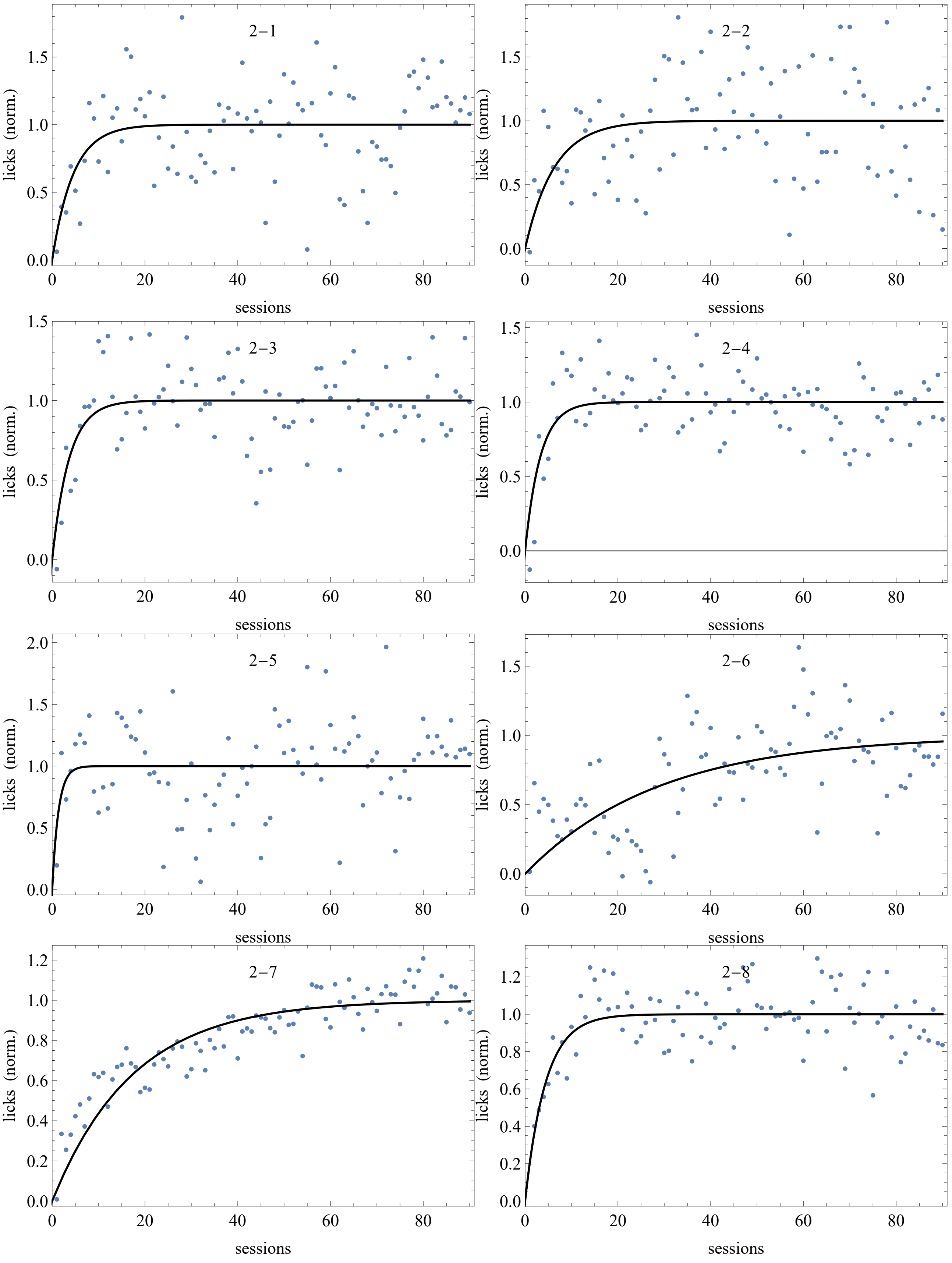

Figure S7. Individual normalized data (CS-licks minus US) of Group 2 with the best RW-model fit. Sessions are on the *horizontal axis*, the normalized response is on the *vertical axis*.

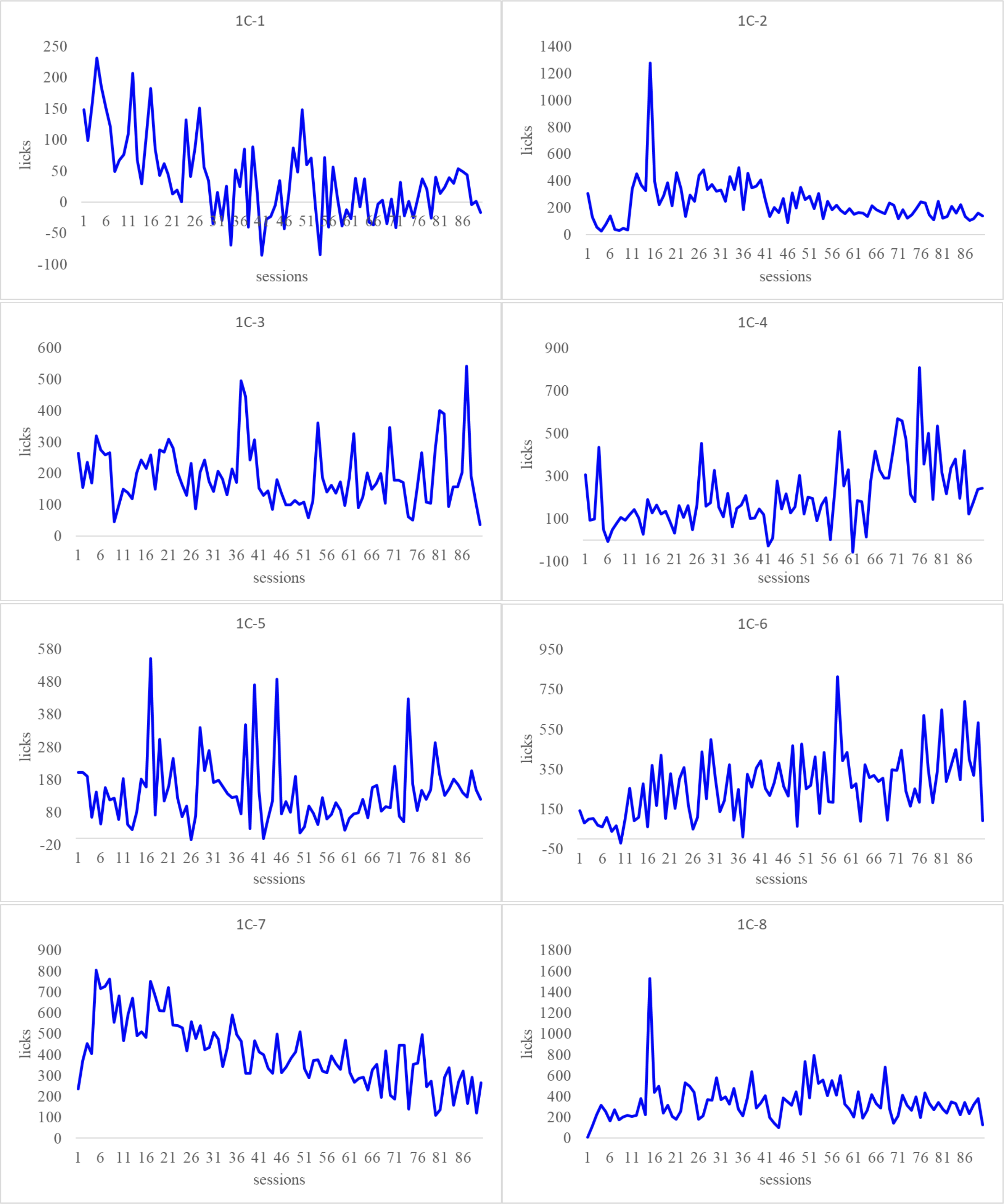

Figure S8. Individual raw data of Group 1C: sum of pre-CS, CS, and post-CS licks, minus the number of US. Sessions are on the *horizontal axis*, the response is on the *vertical axis*. The data of the control groups are not normalized (for instance, with respect to the total average) because this step would not bring any useful information.

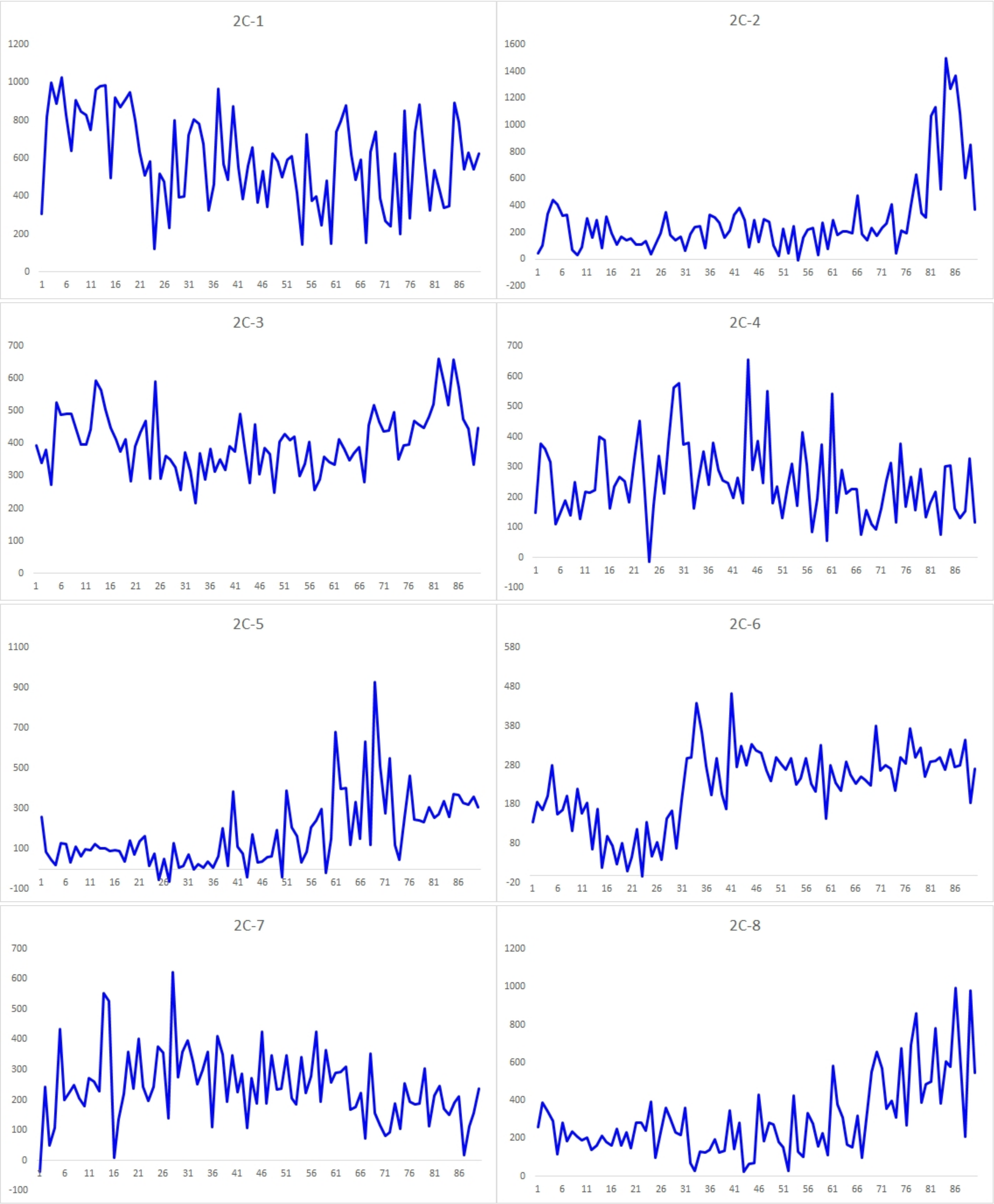

Figure S9. Individual raw data of Group 2C: sum of pre-CS, CS, and post-CS licks, minus the number of US. Sessions are on the *horizontal axis*, the response is on the *vertical axis*.

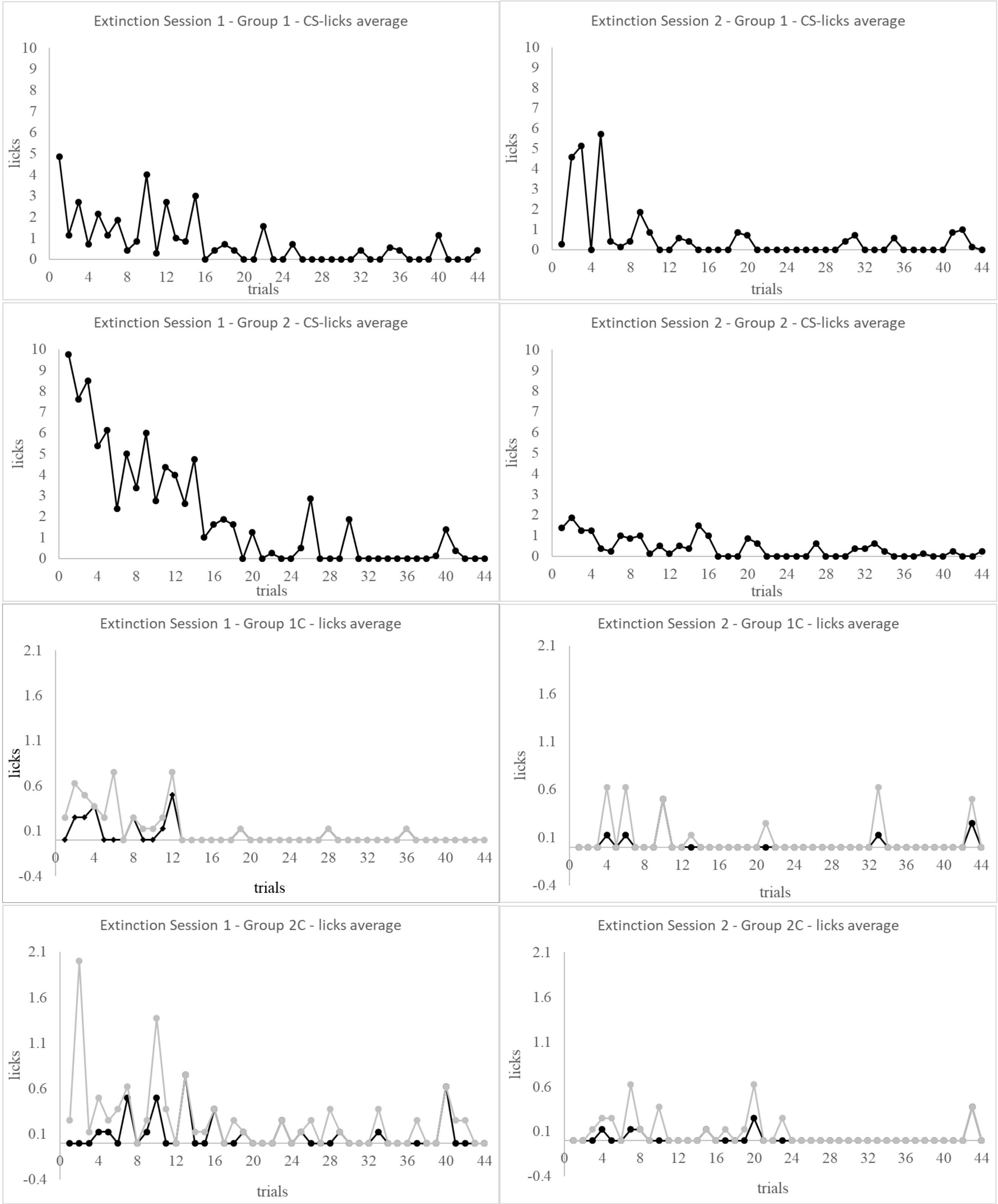

Figure S10. Average of raw (non-normalized) data of the two extinction sessions for experimental and control groups. The black trend is the licks during the CS, while the light gray trend in control plots is the total number of licks 10 s before, during, and 10 s after the CS. Trials are on the *horizontal axis*, the response is on the *vertical axis*.

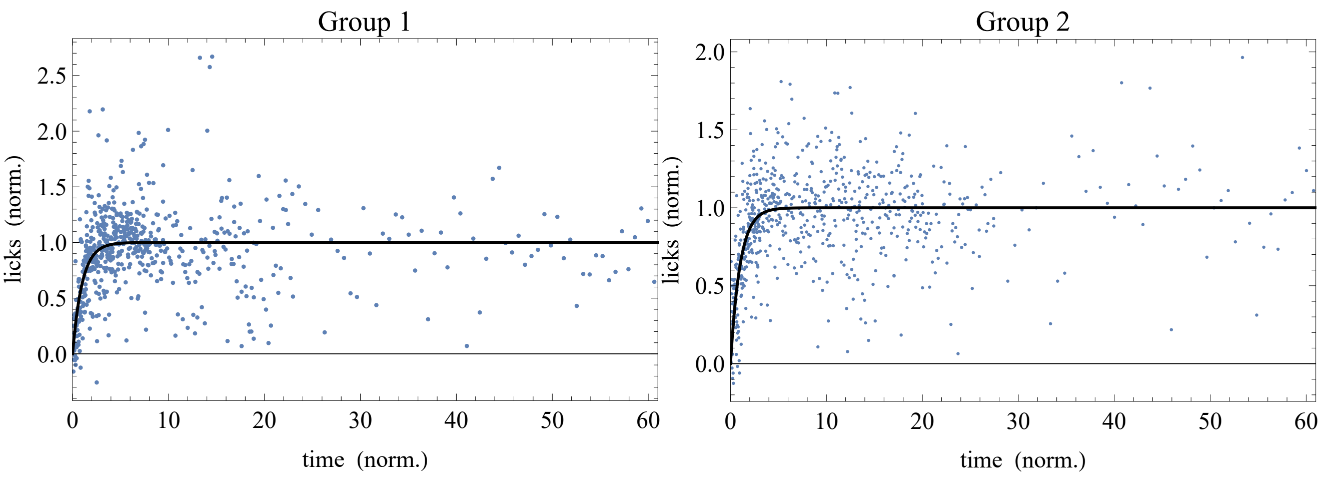

Figure S11. Best fit of doubly normalized data (i.e., with normalized asymptote and normalized time) for Group 1 (left) and 2 (right).

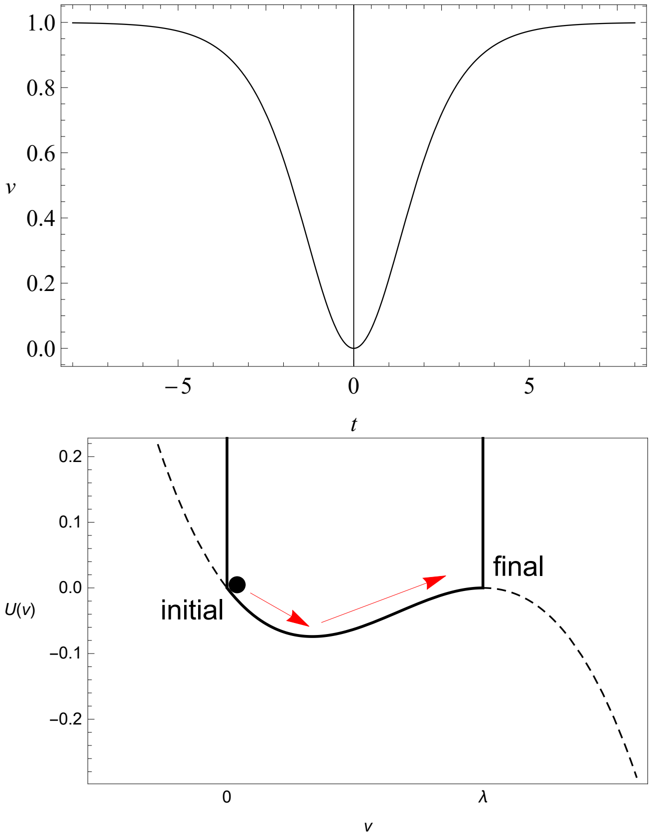

Figure S12. Top panel: the solution (S21a) of Mackintosh conditioning model (S25) for $c = \lambda = 1$, $\beta = 1$ and $\gamma = 1/2$. Bottom panel: the potential $U(v)$ (S24) for $c = \lambda = 1$ and $\beta\gamma = 1/2$. The particle rolling down the potential represents the change in the associative strength. The direction of "motion" in excitatory conditioning is represented by a red arrow.

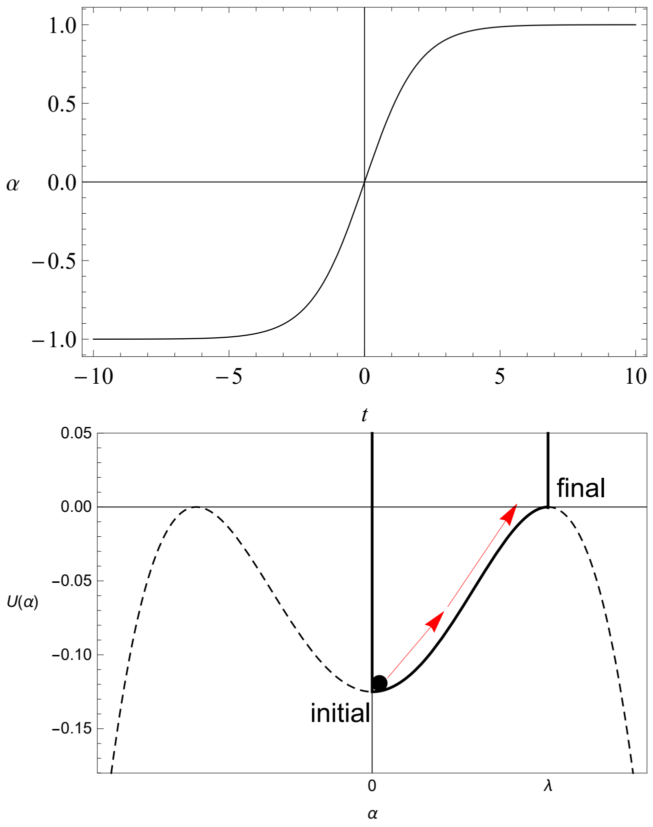

Figure S13. Top panel: the solution (S21b) of Mackintosh conditioning model (S29) for $c = \lambda = 1$, $\beta = 1$ and $\gamma = 1/2$. Bottom panel: the potential $U(\alpha)$ (S28) for $c = \lambda = 1$ and $\beta\gamma = 1/2$. The particle rolling down the potential represents the change in the salience of the CS. The direction of "motion" in excitatory conditioning is represented by a red arrow.

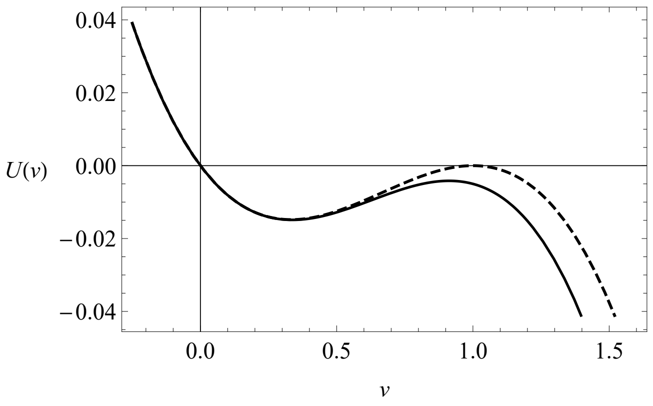

Figure S14. The quartic potential in Eq. (S32) (solid curve) compared with Eq. (S24) (dashed curve), for $\lambda = c = 1$, $\beta = 1$ and $\gamma = 0.1$.

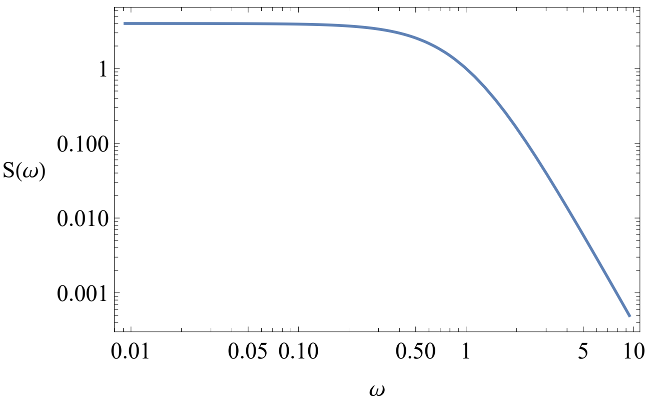

Figure S15. Power spectral density (S35) of the ideal learning curve given by (S2), for $\alpha\beta = 1 = \lambda$.

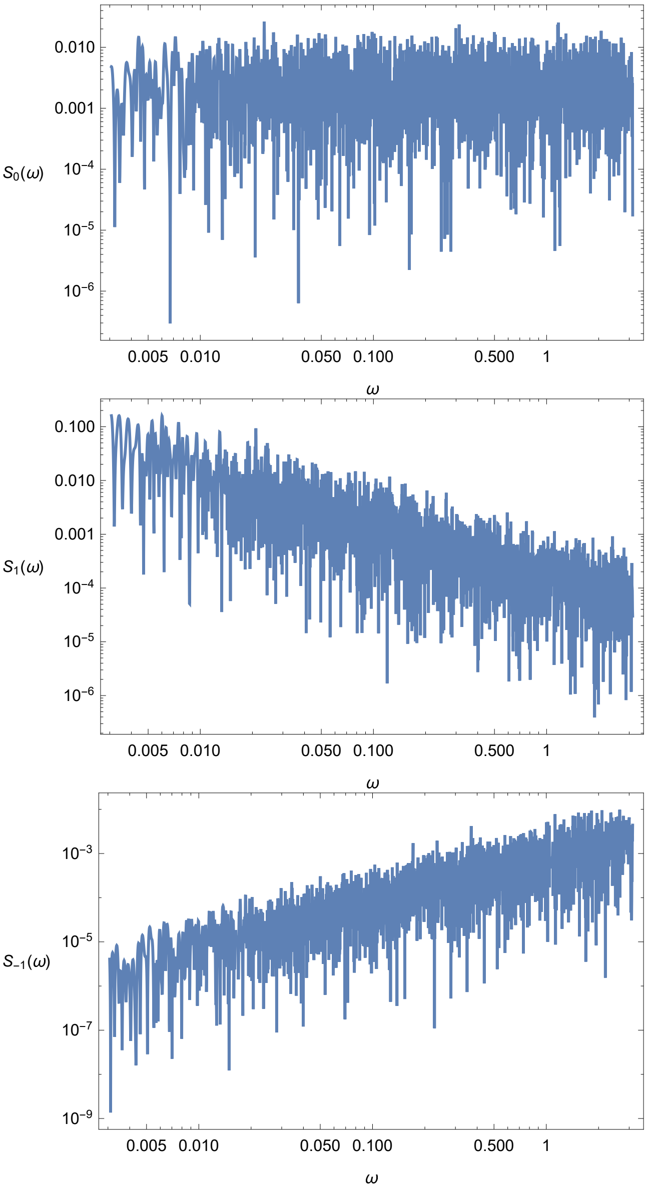

Figure S16. Power spectral density (S36) of white noise (top, $a = 0$), pink noise (middle, $a = 1$), and blue noise (bottom, $a = -1$), simulated with the AudioGenerator of Mathematica.

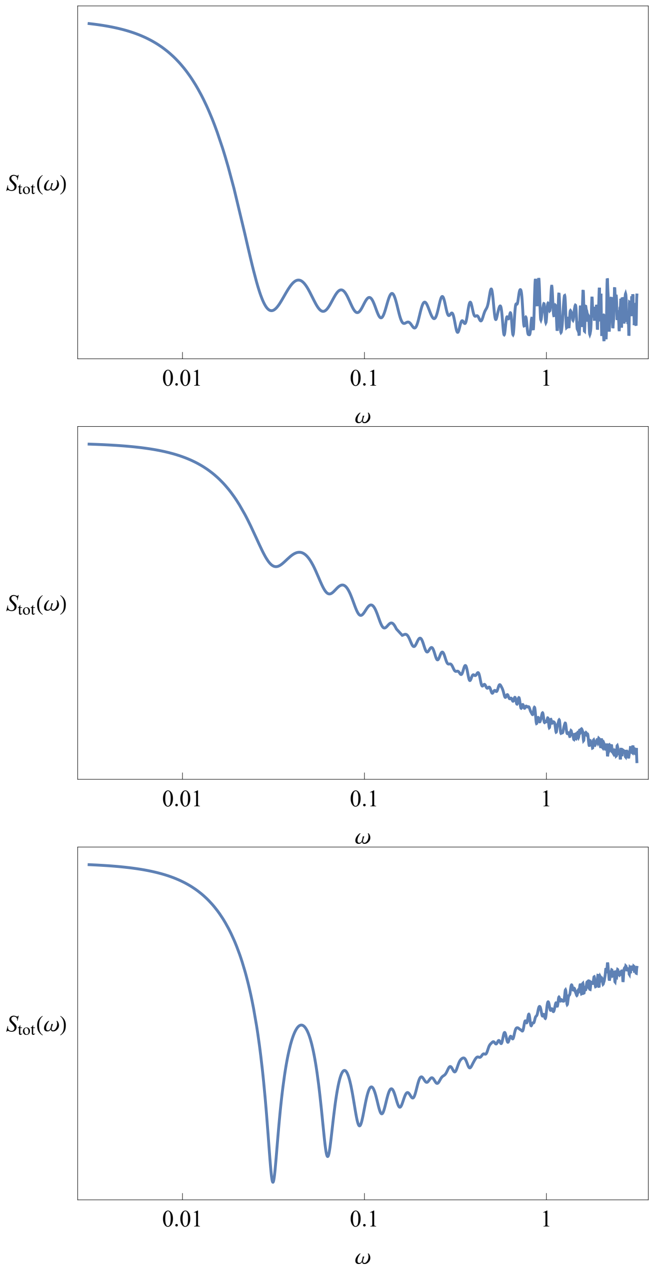

Figure S17. Total power spectral density $S_{\rm tot}(\omega) = S(\omega) + S_a(\omega)$ with white noise (top, $a = 0$), pink noise (middle, $a = 1$), or blue noise (bottom, $a = -1$), simulated with Mathematica. Here $\alpha\beta = 1 = \lambda$ and we used a sample size of 200 data points.

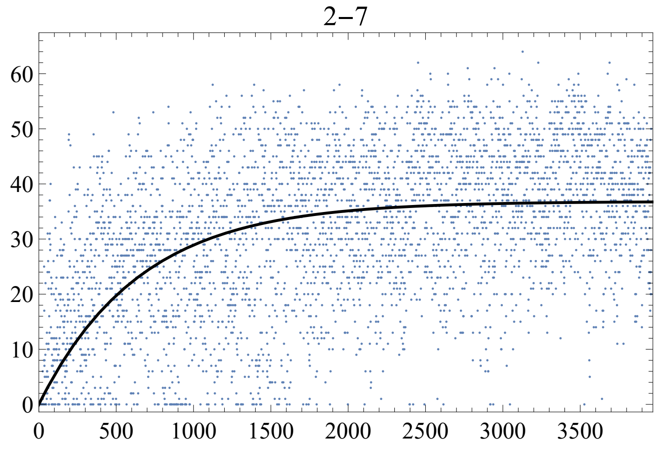

Figure S18. Trial-by-trial data of subject 2-7.

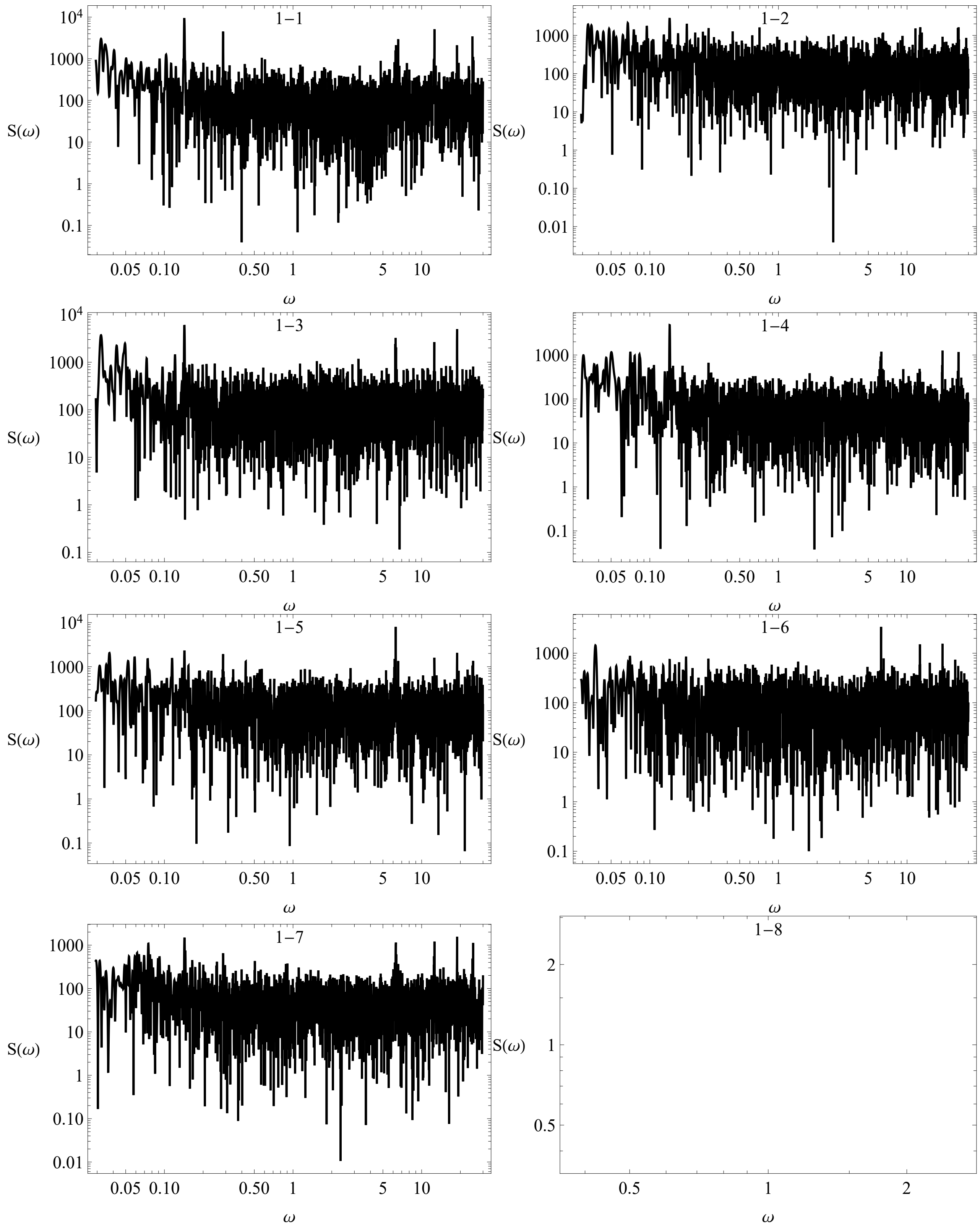

Figure S19. Power spectral density of trial-by-trial data of Group 1. The onset of the spectra on the left agrees with the signal predicted by the RW model, while the noise band at higher frequencies extends indefinitely to the right. Subject 1-8 was removed from the analysis.

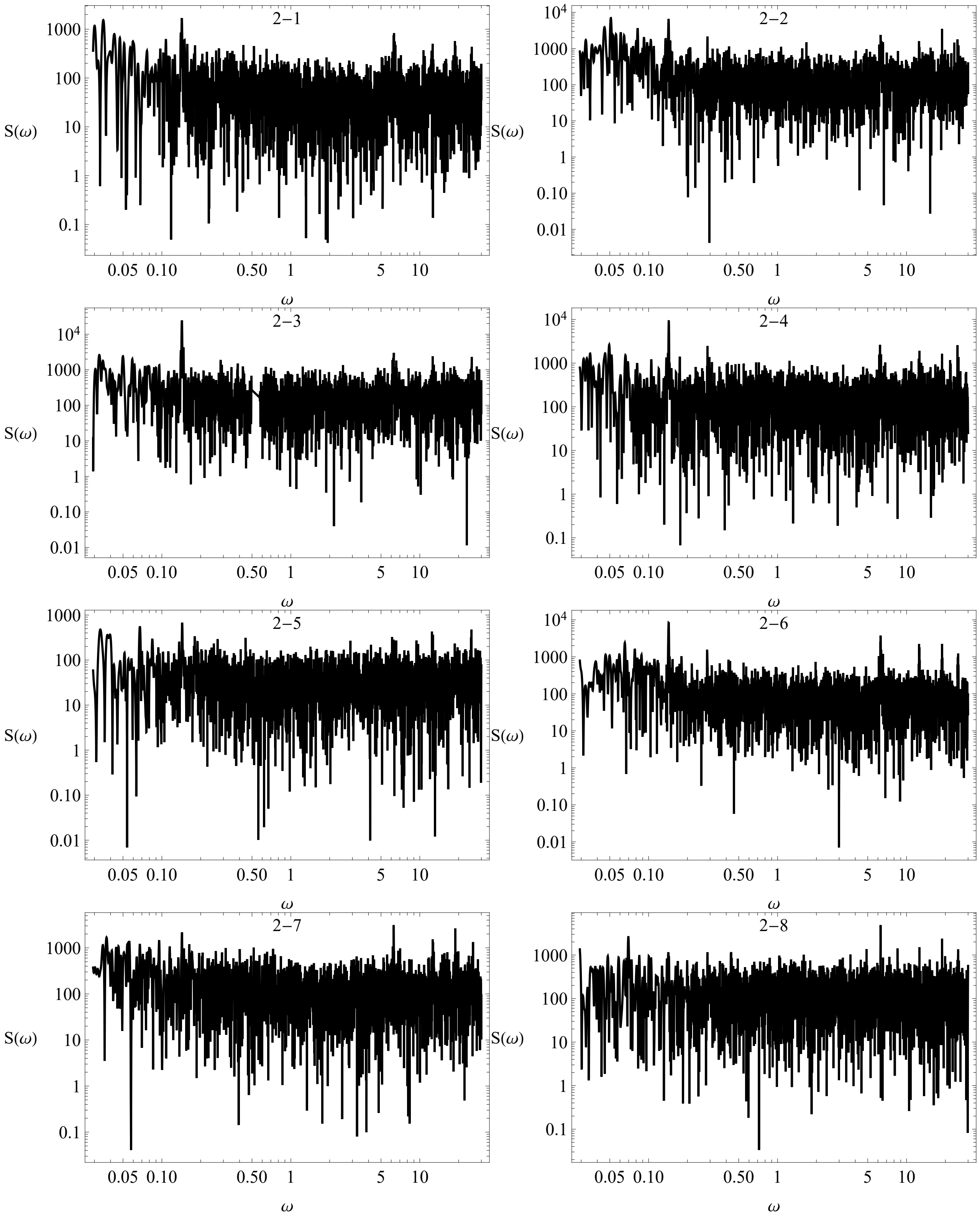

Figure S20. Power spectral density of trial-by-trial data of Group 2. The onset of the spectra on the left agrees with the signal predicted by the RW model, while the noise band at higher frequencies extends indefinitely to the right.

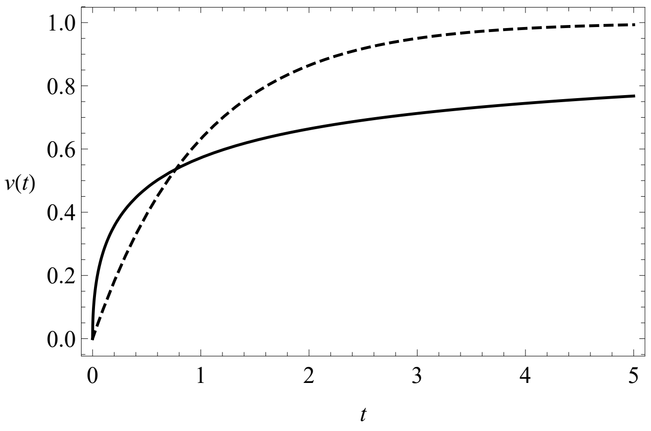

Figure S21. Learning curve (S39) (solid) with $\gamma = 0.5$, compared with the RW curve (S2) (dashed), for $\lambda = 1 = \alpha\beta$.

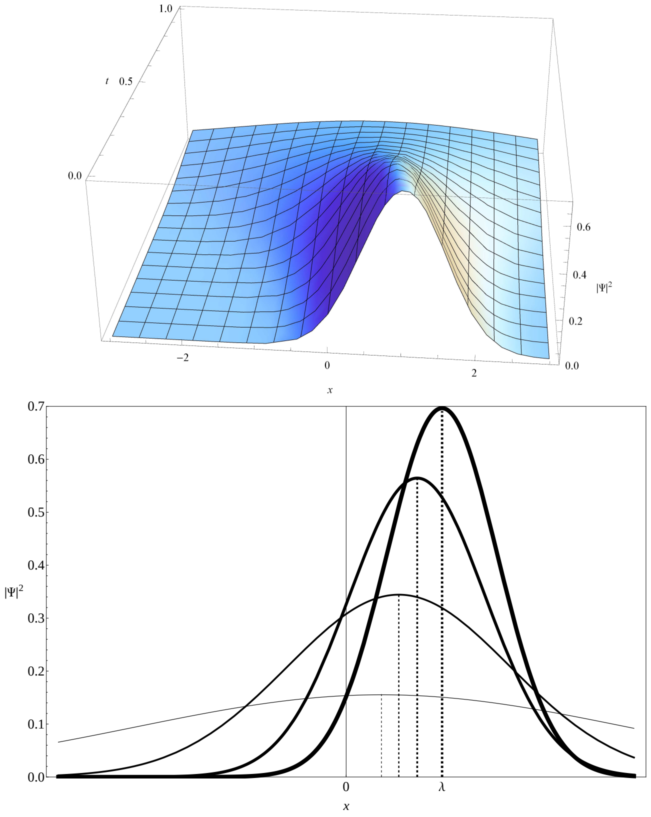

Figure S22. Top: the probability density (S66) for the RW model with $\lambda = 1$, $\Omega = 1$ and $b = 0.9$, as a function of $x = \lambda - v$ and t. Bottom: snapshots of the probability density at $t = 0, 0.3, 0.6, 1$ (decreasing thickness) for the same values of the parameters. Dashed lines indicate the position of the peak $x(t)$ [Eq. (S48) with $x_0 = -p_0 = \lambda$].

	**1-1**	**1-2**	**1-3**	**1-4**
λ_{average}	332	488	860	426
$\lambda_{\text{best fit}}$	299	565	869	425
$(\alpha\beta)_{\text{best fit}}$	0.26 ± 0.16	0.05 ± 0.01	0.11 ± 0.02	0.67 ± 0.38
	**1-5**	**1-6**	**1-7**	**1-8**
λ_{average}	856	493	336	
$\lambda_{\text{best fit}}$	889	503	331	
$(\alpha\beta)_{\text{best fit}}$	0.09 ± 0.01	0.09 ± 0.02	0.21 ± 0.09	
	**2-1**	**2-2**	**2-3**	**2-4**
λ_{average}	361	599	769	762
$\lambda_{\text{best fit}}$	362	604	775	778
$(\alpha\beta)_{\text{best fit}}$	0.22 ± 0.07	0.16 ± 0.06	0.27 ± 0.07	0.30 ± 0.07
	**2-5**	**2-6**	**2-7**	**2-8**
λ_{average}	225	546	1417	815
$\lambda_{\text{best fit}}$	234	682	1553	827
$(\alpha\beta)_{\text{best fit}}$	0.74 ± 0.47	0.03 ± 0.01	0.06 ± 0.00	0.23 ± 0.03

Table S1. Asymptote λ (average number of licks during the CS minus number of US, after acquisition) determined by the averaging and best-fit procedures described in the text for session-by-session data. The association rate $\alpha\beta$ obtained by the best fit is also shown.

	**1-1**	**1-2**	**1-3**	**1-4**
λ	8.9 ± 0.2	14.7 ± 0.3	21.8 ± 0.2	11.9 ± 0.1
$\alpha\beta$	0.00832 ± 0.00213	0.00155 ± 0.00015	0.00298 ± 0.00022	0.02458 ± 0.00595
σ	12.1	13.5	12.8	9.1
	**1-5**	**1-6**	**1-7**	**1-8**
λ	22.2 ± 0.3	13.4 ± 0.2	9.7 ± 0.1	
$\alpha\beta$	0.00273 ± 0.00018	0.00264 ± 0.00025	0.00677 ± 0.00097	
σ	12.4	10.5	8.1	
	**2-1**	**2-2**	**2-3**	**2-4**
λ	10.3 ± 0.1	15.7 ± 0.2	19.7 ± 0.3	19.8 ± 0.2
$\alpha\beta$	0.00688 ± 0.00095	0.00696 ± 0.00111	0.00793 ± 0.00115	0.00865 ± 0.00119
σ	8.2	14.3	15.5	14.2
	**2-5**	**2-6**	**2-7**	**2-8**
λ	7.5 ± 0.1	17.0 ± 0.4	36.8 ± 0.3	21.0 ± 0.2
$\alpha\beta$	0.77470 ± 1.21695	0.00103 ± 0.00008	0.00154 ± 0.00006	0.00632 ± 0.00066
σ	6.6	11.8	12.7	13.2

Table S2. RW-model best-fit parameters of trial-by-trial data in our experiment. σ is the estimated standard error.

$S_a(\omega) = \omega^{-a}$	**1-1**	**1-2**	**1-3**	**1-4**
a	0.016 ± 0.011	-0.003 ± 0.010	-0.016 ± 0.014	0.003 ± 0.010
Color	white*	white	white*	white
	**1-5**	**1-6**	**1-7**	**1-8**
a	-0.011 ± 0.016	-0.007 ± 0.010	-0.010 ± 0.013	
Color	white	white	white	
	**2-1**	**2-2**	**2-3**	**2-4**
a	0.010 ± 0.010	-0.001 ± 0.010	0.021 ± 0.012	0.007 ± 0.008
Color	white	white	white*	white
	**2-5**	**2-6**	**2-7**	**2-8**
a	0.002 ± 0.007	-0.009 ± 0.023	-0.044 ± 0.051	-0.006 ± 0.008
Color	white	white	white	white

Table S3. Type of noise in the noise spectrum $S_a(\omega)$ of individual trial-by-trial data in the long experiment, analyzed between $\omega = 0.1$ and $\omega = 30$. The error is one standard deviation. Asterisks denote conclusions valid within two standard deviations.

**Subject**	**CR10**	**CR30**	**PR10**	**PR30**
**1**	-0.09 ± 0.13	-0.03 ± 0.04	-0.02 ± 0.12	-0.06 ± 0.08
**2**	$+0.00 \pm 0.13$	-0.03 ± 0.07	-0.08 ± 0.06	-0.09 ± 0.19
**3**	-0.00 ± 0.05	-0.03 ± 0.05	-0.03 ± 0.04	-0.03 ± 0.04
**4**	$+0.01 \pm 0.17$	-0.08 ± 0.12	-0.08 ± 0.07	-0.09 ± 0.15
**5**	-0.01 ± 0.06	-0.06 ± 0.07	-0.06 ± 0.13	-0.06 ± 0.08
**6**	-0.05 ± 0.06	-0.05 ± 0.03	-0.08 ± 0.06	-0.03 ± 0.03
**7**	-0.01 ± 0.14	-0.06 ± 0.07	-0.00 ± 0.03	-0.07 ± 0.12
**8**	-0.04 ± 0.04	-0.03 ± 0.02	-0.05 ± 0.05	-0.05 ± 0.04
**9**	-0.01 ± 0.13	-0.07 ± 0.11	-0.05 ± 0.06	-0.08 ± 0.17
**10**	$+0.00 \pm 0.07$	-0.05 ± 0.06	-0.06 ± 0.05	-0.06 ± 0.10
**11**	-0.02 ± 0.05	-0.05 ± 0.05	-0.02 ± 0.09	-0.05 ± 0.06
**12**	$+0.02 \pm 0.09$	-0.09 ± 0.11	-0.07 ± 0.08	-0.08 ± 0.16
**13**	-0.00 ± 0.13	-0.08 ± 0.09	-0.06 ± 0.04	-0.06 ± 0.10
**14**	-0.02 ± 0.05	-0.06 ± 0.06	-0.06 ± 0.07	-0.07 ± 0.11
**15**	-0.03 ± 0.04	-0.03 ± 0.03	-0.06 ± 0.07	-0.07 ± 0.05
**16**	-0.04 ± 0.07	-0.03 ± 0.04	-0.03 ± 0.06	-0.05 ± 0.06
**Average**	-0.02 ± 0.10	-0.05 ± 0.07	-0.05 ± 0.07	-0.06 ± 0.11
**Color**	white	white	white	white

Table S4. Best fit of individual trial-by-trial data of Experiment 2 of Harris et al. (2015) with the noise spectrum (S36) in the range $1 < \omega < 60$. The value of the parameter a with the fit error is shown for each subject. Some subjects have a non-white noise at the 1σ-level but all these subjects have white noise at the 2σ-level. In the group average, the error is the propagated one.

		**CR10**	**CR30**	**PR10**	**PR30**
	λ	1.46	1.03	1.08	0.72
RW	σ	0.32	0.29	0.33	0.27
	σ/λ	0.22	0.29	0.31	0.38
	λ	0.61	0.58	0.52	0.54
Oscillations	σ	0.30	0.26	0.32	0.28
	σ/λ	0.48	0.45	0.61	0.52

Table S5. Average value of the asymptote of learning and of data dispersion in Experiment 2 of Harris et al. (2015).

Subject	Group 1	Group 2
1	2.4	0.9
2	0.6	2.8
3	1.0	3.8
4	4.1	3.5
5	0.8	*67.5*
6	0.6	0.3
7	0.9	0.5
8		2.2
Average	1.5 ± 1.2	10.2 ± 21.7
Corr. average		2.0 ± 1.3

Table S6. Estimate (S77) of $\bar{h}$ for the individual subjects and in average for our long experiment. In the corrected average of Group 2, the datum of subject 2-5 (in italics) has been removed.

Subject	CR10	CR30	PR10	PR30
1				0.0563
2	0.0055	0.0040	0.0009	
3	0.0278	0.0045	0.0069	0.0029
4	0.0016			
5				0.0229
6				
7			0.0014	
8		0.0784	0.0140	
9	0.0027	0.0015	0.0006	0.0004
10	0.0005	0.0013	0.0002	0.0004
11				
12	0.0017			
13	0.0025	0.0022	0.0017	
14				
15		0.0091	0.0024	
16		0.0062	0.0043	
Average	0.006 ± 0.009	0.013 ± 0.025	0.004 ± 0.004	0.017 ± 0.022

Table S7. Estimate (S77) of $\bar{h}$ for the individual subjects and in average for Experiment 2 of Harris et al. (2015).
